# An Engineered Nano‐Bridging Strategy Remodels the Immune Microenvironment of Esophageal Squamous Cell Carcinoma via STING Pathway Activation

**DOI:** 10.1002/mco2.70828

**Published:** 2026-06-13

**Authors:** Chaofan Huang, Xin Tang, Yixiao Zhao, Dafu Xu, Congyong Sun, Chenlong Wang, Fei Xie, Fangyi Xu, Chao Luo, Li Zhang, Qilong Wang

**Affiliations:** ^1^ Department of Central Laboratory The Affiliated Huaian No. 1 People's Hospital of Nanjing Medical University, Northern, Nanjing Medical University Huai'an Jiangsu Province China; ^2^ Department of Thoracic Surgery The Affiliated Huaian No. 1 People's Hospital of Nanjing Medical University Huaian China

**Keywords:** esophageal squamous cell carcinoma, nano‐immunotherapy, phagocytosis, STING, tumor‐associated macrophages

## Abstract

Restoring macrophage‐mediated phagocytosis represents a promising strategy for enhancing antitumor innate immunity. However, the highly immunosuppressive tumor immune microenvironment (TIME) of esophageal squamous cell carcinoma (ESCC) limits macrophage infiltration, impairs effector function, and constrains durable responses to immunotherapy. Here, we developed a multifunctional nanoplatform, HA1@diABZI–HMGB1, integrating the ESCC‐targeting aptamer HA1, the damage‐associated molecular pattern protein HMGB1, and the STING agonist diABZI to remodel the TIME through coordinated immune regulation. In this system, HA1 was designed to improve tumor‐cell recognition and tumor‐site accumulation, whereas HMGB1 was introduced to promote macrophage–tumor cell interaction and facilitate phagocytic engagement. Meanwhile, intracellular delivery of diABZI activated STING signaling, enhanced proinflammatory cytokine production, and promoted the repolarization of tumor‐associated macrophages toward an M1‐like phenotype. In ESCC models, HA1@diABZI–HMGB1 enhanced macrophage phagocytosis, reduced M2‐like macrophage accumulation, increased dendritic‐cell maturation, promoted cytotoxic T‐lymphocyte infiltration, and suppressed tumor progression without apparent systemic toxicity. Mechanistically, this integrated strategy combined tumor‐targeted anchoring, immune‐cell bridging, and innate immune activation within a single nanosystem. Together, these findings support the potential of this nanoplatform to remodel the ESCC immune microenvironment at the preclinical level and provide a basis for further investigation of macrophage‐centered immunomodulatory strategies.

## Graphical abstract

HA1@diABZI–HMGB1 serves as a synergistic immunomodulator for dismantling the immunosuppressive microenvironment of esophageal squamous cell carcinoma (ESCC), it integrates ESCC‐targeted recognition, HMGB1‐mediated macrophage–tumor cell bridging, and diABZI‐driven STING activation. This nanoplatform enhances macrophage phagocytosis, promotes M2‐to‐M1 repolarization, and improves the immunosuppressive ESCC microenvironment, providing a macrophage‐centered nano‐immunotherapeutic strategy for solid tumors.

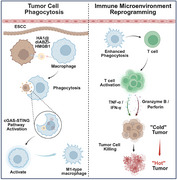

## 1 Introduction

Esophageal cancer is a highly lethal malignancy of the gastrointestinal tract that poses a formidable challenge to global public health. According to recent statistics, more than 511,000 new cases and approximately 445,000 deaths occur annually worldwide [1]. The burden of this disease is particularly pronounced in China, which accounts for more than 40% of the global caseload, with esophageal squamous cell carcinoma (ESCC) as the predominant histological subtype. Despite the available multimodal treatment regimens encompassing surgery and chemoradiotherapy, the prognosis of patients with ESCC remains poor, with a 5‐year survival rate of <20% [2]. Immune checkpoint inhibitors have reshaped the therapeutic landscape [3, 4]; however, the clinical benefits are severely limited by the highly immunosuppressive nature of the ESCC tumor microenvironment (TME), which renders most patients immunologically tolerant or nonresponsive [5]. Therefore, strategies that can transform the suppressive ESCC TME into an immunologically active state are urgently needed.

The ESCC TME consists of heterogeneous tumor, immune, stromal, and vascular components that collectively determine the initiation, magnitude, and durability of antitumor immune responses. In solid tumors, immune resistance is not solely determined by the presence or absence of effector cells, but also by whether these cells can infiltrate tumor tissues, maintain functional activity, and establish productive interactions with malignant cells. While blockade strategies targeting “don't eat me” signals (e.g., CD47–SIRPα) or enhancing “eat me” signals (e.g., calreticulin exposure) have achieved breakthroughs in hematological malignancies [6, 7, 8, 9]. their efficacy in solid tumors is fundamentally limited by the lack of effective physical contact between immune and tumor cells due to spatial restrictions [10]. These observations suggest that effective immunotherapy for ESCC should not only modulate immune signaling pathways, but also help overcome the physical and spatial constraints that restrict immune‐cell engagement within the tumor architecture.

Tumor‐associated macrophages (TAMs) constitute the most abundant population of infiltrating immune cells within the ESCC microenvironment and are key drivers of tumor progression and therapeutic resistance [11, 12]. The functional plasticity of TAMs renders them ideal targets for immunotherapy. Tumor cells evade macrophage‐mediated phagocytosis by upregulating “don't eat me” signals [13, 14, 15]; however, the accumulation of M2‐like TAMs constructs a robust immune barrier by secreting suppressive cytokines, inducing angiogenesis, and inhibiting T‐cell cytotoxicity, all of which directly correlate with poor patient outcomes [16, 17]. Conversely, macrophages with an M1‐like phenotype can support inflammatory cytokine production, antigen presentation, tumor‐cell killing, and adaptive immune activation. Therefore, a “two‐pronged” strategy, concurrently relieving the phagocytic brake and reprogramming TAMs to an antitumor M1 phenotype, is a pivotal direction for overcoming immune tolerance in ESCC.

The cGAS–STING pathway provides a mechanistic opportunity to connect innate immune activation with broader antitumor immune remodeling. Activation of this pathway can induce Type I interferon (IFN‐I)‐related inflammatory responses, promote antigen‐presenting cell activation, and support the recruitment and activation of effector lymphocytes [18]. In addition, STING activation may directly influence macrophage polarization and thereby contribute to the conversion of an immunosuppressive TME into an immune‐active state [19]. Nevertheless, small‐molecule STING agonists face several delivery‐related barriers, including limited tumor accumulation, inefficient intracellular delivery, rapid systemic clearance, and potential off‐target toxicity [20]. These limitations are particularly relevant in ESCC, where the immunosuppressive and spatially restricted TME may prevent STING agonist monotherapy from achieving sufficient immune activation. Thus, a delivery system that improves STING agonist bioavailability while simultaneously promoting macrophage–tumor cell interaction may provide a more rational therapeutic strategy.

To address these challenges, we propose a multimodal nano‐strategy designed to synergistically potentiate macrophage‐mediated antitumor immunity. We constructed a functionalized biomimetic nanoparticle (HA1@diABZI–HMGB1) that integrates an ESCC‐targeting aptamer (HA1) with the damage‐associated molecular pattern protein, HMGB1, to create a dual‐targeting “immune bridging” system. This design was developed to bring macrophages into closer contact with tumor cells and thereby support macrophage‐mediated phagocytosis. At the same time, it enables the delivery of the STING agonist diABZI, which may activate the intracellular cGAS–STING pathway after cellular uptake. By enhancing phagocytic activity and promoting TAM repolarization toward an M1‐like phenotype, this multifunctional nanoplatform may further contribute to T cell‐mediated adaptive immune responses. These effects could help alleviate the immunosuppressive ESCC microenvironment. Through systematic in vitro and in vivo studies, our results support the potential role of this nanosystem in promoting phagocytosis, modulating the immune microenvironment, and inhibiting tumor growth. This study provides a promising nanomedicine‐based strategy for ESCC immunotherapy.

## 2 Results

### 2.1 Screening, Identification, and Targeting Validation of the ESCC‐Targeting Aptamer HA1

**FIGURE 1:**
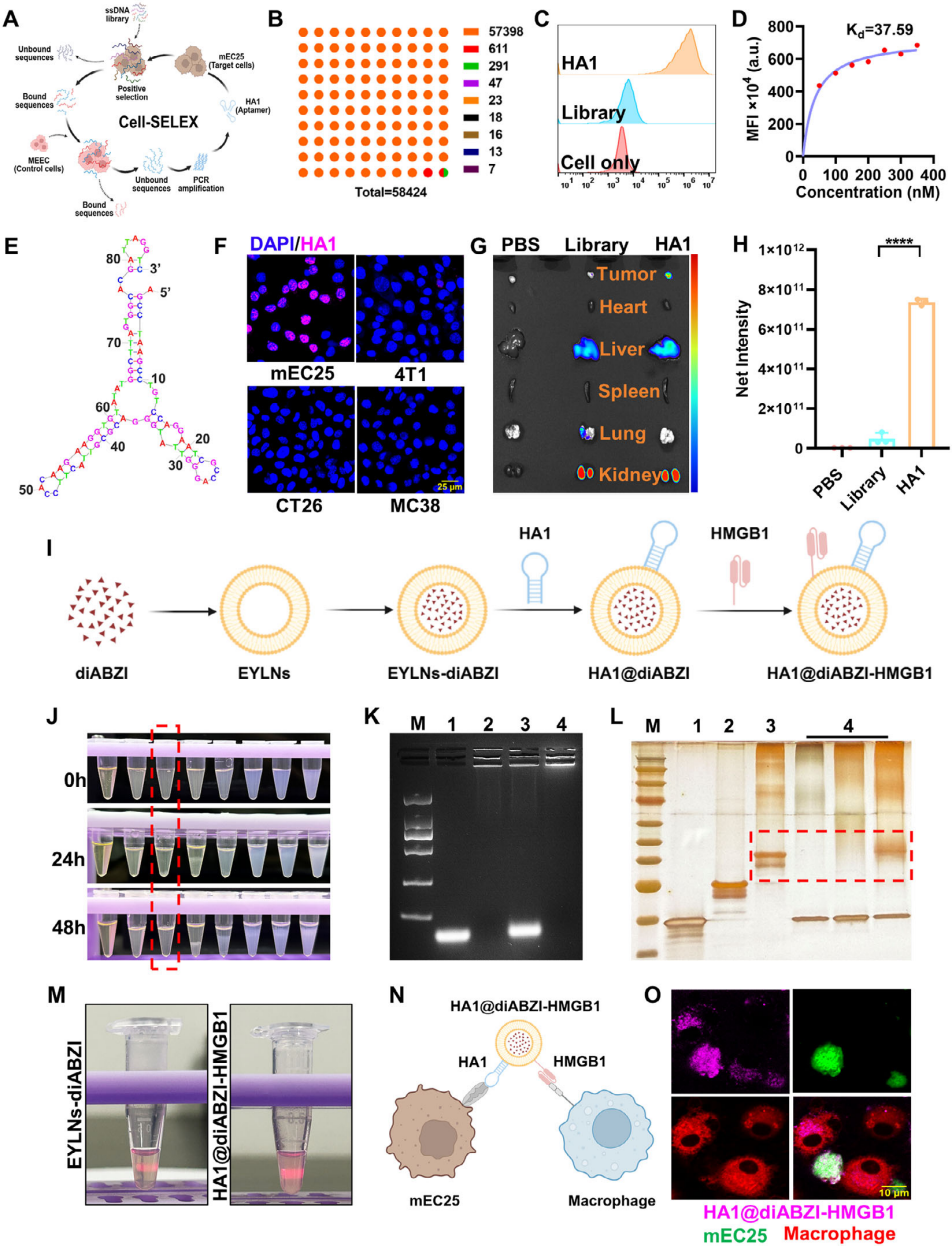
Screening, identification, and specificity verification of ESCC‐targeting aptamer HA1 and preparation of HA1@diABZI–HMGB1. (A) A schematic diagram illustrating the Cell‐SELEX process. (B) The screening library was highly enriched, and the selected aptamer was designated as HA1. (C) Flow cytometry analysis of the candidate aptamer, labeled with FAM, targeting mEC25 cells. (D) Determination of the dissociation constant (*K*_d_) of HA1 with mEC25 cells using flow cytometry. (E) Prediction of the secondary structure of HA1 utilizing RNAfold. (F) Confocal microscopy imaging to assess the colocalization of Cy5‐labeled HA1 with target mEC25 cells and control cell lines. (G) Evaluation of the in vivo targeting capability and biodistribution of Cy5‐labeled HA1 in a subcutaneous tumor model. (H) Quantitative analysis of fluorescence signals at the tumor site. Data are shown as the mean ± SD (*n* = 3), and statistical significance was calculated via a two‐tailed unpaired *t*‐test, ****p < 0.0001. (I) Schematic representation illustrating the synthesis process of HA1@diABZI–HMGB1. (J) Optimization of the ratio between EYLNs and diABZI. (K) Verification of the conjugation between HA1 and EYLNs using 3% agarose gel electrophoresis. M: DNA marker; 1: Free HA1; 2: EYLNs–diABZI; 3: Free HA1 combined with EYLNs–diABZI; 4: HA1@diABZI. (L) Confirmation of HMGB1 incorporation through silver staining. M: Protein marker; 1: HA1; 2: HMGB1; 3: DBCO–HMGB1; 4: HA1@diABZI–HMGB1. (M) Examination of the bright‐field morphology and Tyndall effect of EYLNs–diABZI and HA1@diABZI–HMGB1. (N) Confocal laser scanning microscopy image depicting the interaction of Cy5‐labeled HA1@diABZI–HMGB1 (pink) with PKH26‐labeled BMDM cells (red) and CFSE‐labeled mEC25 cells (green). (O) Schematic depiction of the mechanism by which the HA1@diABZI–HMGB1 nanosystem facilitates the bridging between macrophages and tumor cells.

To endow our nano‐delivery system with active targeting capability against ESCC, we screened high‐affinity nucleic acid aptamers using Cell‐Systematic Evolution of Ligands by Exponential Enrichment (Cell‐SELEX). Mouse ESCC cells (mEC25) were used for positive selection, whereas normal mouse esophageal epithelial cells were used for counter‐selection to eliminate nonspecific sequences. After 20 stringent rounds of binding, elution, and amplification, the enriched ssDNA library was subjected to high‐throughput sequencing (Figure 1A). From 58,424 valid sequences, HA1 was selected as the primary candidate for subsequent validation based on sequence abundance and homology clustering (Figure 1B).

To quantify the binding of HA1 to ESCC cells, we incubated mEC25 cells with FAM‐labeled HA1 and performed flow cytometric analysis. HA1 induced a marked fluorescence shift compared with the randomized library sequence, confirming its specific binding to target cells (Figure 1C). Nonlinear regression fitting yielded an equilibrium dissociation constant (*K*_d_) of 37.59 ± 19.29 nM for the HA1–mEC25 interaction, indicating a high‐binding affinity at the nanomolar level (Figure 1D). Furthermore, secondary structure prediction using mFold showed that HA1 formed a stable stem–loop structure, which may support target recognition (Figure 1E).

We next assessed the targeting selectivity of HA1 using Cy5‐labeled probes in different tumor cell lines. Confocal laser scanning microscopy (CLSM) showed strong Cy5–HA1 fluorescence in mEC25 cells, whereas only negligible signals were detected in nontarget breast cancer cells (4T1) and colon cancer cells (CT26 and MC38) (Figure 1F). These findings indicate that HA1 preferentially recognizes ESCC cells over the tested non‐ESCC tumor cells.

Based on in vitro results, we evaluated the in vivo targeting potential of HA1 in mEC25 subcutaneous mouse models. Following the tail vein injection of Cy5–HA1, near‐infrared fluorescence imaging showed predominant HA1 enrichment in the tumor region, with tumor fluorescence intensity exceeding that of surrounding normal tissues over time (Figure 1G,H). Therefore, HA1 appeared to retain targeting activity in vivo and support its use for constructing HA1‐functionalized ESCC‐targeted nanoplatforms.

### 2.2 Biomimetic Design and Construction of the Multifunctional Bridging Nanoparticle HA1@diABZI–HMGB1

To improve ESCC targeting and immune microenvironment modulation, we developed a multifunctional biomimetic nanoplatform, HA1@diABZI–HMGB1, designed to integrate molecular bridging with STING‐mediated immune activation. In this system, the nanoparticle core encapsulated the small‐molecule STING agonist diABZI, whereas the surface was functionalized with the ESCC‐targeting aptamer HA1 and the damage‐associated molecular pattern protein HMGB1. This design was intended to enhance tumor‐cell recognition, promote macrophage–tumor cell interaction, and support macrophage‐mediated phagocytosis (Figure 1I).

We first optimized the drug‐loaded lipid core by screening the ratio between egg yolk‐derived lipid nanoparticles (EYLNs) and diABZI. Because increasing the drug proportion led to phase separation, the optimal EYLNs‐to‐diABZI molar ratio was established at 20:0.125, corresponding to a mass ratio of 3 mg:22.15 µg. Under this condition, the nanosystem maintained a uniform and stable dispersion for 48 h without precipitation or stratification (Figure 1J). To enable HA1 surface assembly, polyethyleneimine (PEI) was introduced as an electrostatic anchor. Agarose gel retardation assays showed that 82.5 µg of PEI completely complexed 3 nmol of HA1 (Figure S1A,B), and HA1@diABZI displayed reduced electrophoretic mobility compared with free HA1 or the physical mixture, supporting successful HA1 association with the liposome surface (Figure 1K).

HMGB1 was then conjugated to the nanoparticle surface through copper‐free click chemistry by coupling DBCO‐modified HMGB1 with azide‐functionalized lipids incorporated into the lipid bilayer. SDS‐PAGE and silver staining confirmed successful HMGB1 modification, as indicated by covalent linkage bands between HMGB1 and lipid molecules (Figure 1L). The final HA1@diABZI–HMGB1 nanoparticles appeared as a uniform pale‐yellow emulsion and showed a clear Tyndall effect, indicatingexcellent colloidal dispersion (Figure 1M). CLSM imaging further showed fluorescent contact signals between macrophages and ESCC cells after treatment with HA1@diABZI–HMGB1 (Figure 1N,O), suggesting that the system may facilitate macrophage–tumor cell interactions for subsequent immune activation.

### 2.3 Physicochemical Characterization of HA1@diABZI–HMGB1

**FIGURE 2:**
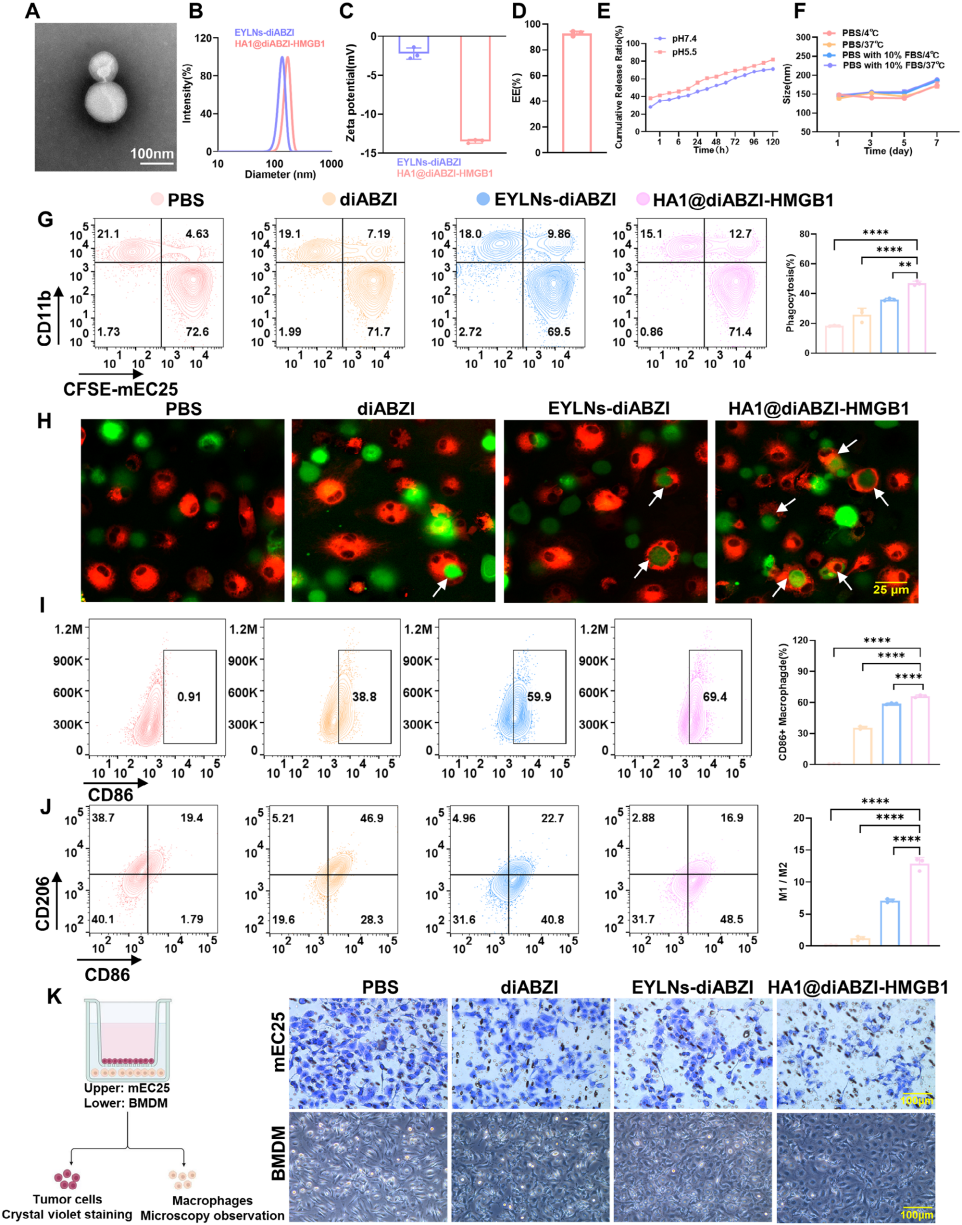
Characterization of HA1@diABZI–HMGB1 and its effects on enhancing macrophage phagocytosis and promoting M1‐like polarization. (A) Representative transmission electron microscopy (TEM) image of HA1@diABZI–HMGB1 (scale bar: 100 nm). (B) Particle size distribution of EYLNs–diABZI and HA1@diABZI–HMGB1 analyzed by dynamic light scattering (DLS). (C) Surface Zeta potential of EYLNs–diABZI and HA1@diABZI–HMGB1. (D) Encapsulation efficiency of diABZI by HA1@diABZI–HMGB1. (E). In vitro drug release curves of HA1@diABZI–HMGB1 in different PBS buffer solutions (pH 7.4 and pH 5.5). (F) Stability of HA1@diABZI–HMGB1 tested by measuring particle size at 4 and 37°C in PBS and PBS containing 10% FBS. (G) Representative flow cytometry plots illustrate the phagocytosis of mEC25 tumor cells by bone marrow‐derived macrophages (BMDMs) following various treatments. The mEC25 cells were labeled with CFSE, while the macrophages were stained with CD11b. (H) Confocal laser scanning microscopy images depict mEC25 tumor cells engulfed by BMDMs after different treatments, with mEC25 cells labeled in green using CFSE and BMDMs labeled in red using PKH26. The scale bar represents 25 µm. (I) Flow cytometric analysis was conducted to evaluate CD86 expression on BMDMs following various treatments. (J) A flow cytometry‐based assessment was performed to examine the reversal of IL‐4‐induced M2‐like polarization of BMDMs by different treatments. The quantitative analysis of the M1/M2 index, defined as CD86^+^CD206^−^ M1‐like versus CD86^−^CD206^+^ M2‐like macrophages, is presented. (K) Microscopic images of crystal violet‐stained mEC25 tumor cells and morphological analysis of macrophages were obtained after coculture with BMDMs in a Transwell system under various treatments. All data are shown as the mean ± SD (*n* = 3). Statistical significance was calculated through one‐way ANOVA using the Tukey's posttest. **p < 0.01, ****p < 0.0001.

To evaluate the pharmaceutical properties of HA1@diABZI–HMGB1, we characterized its morphology, particle size, zeta potential, encapsulation efficiency, stability, and drug‐release behavior. Transmission electron microscopy (TEM) images showed that the nanoparticles exhibited a uniform spherical structure with lipid bilayer features (Figures 2A and S1C). Compared with EYLNs–diABZI, HA1@diABZI–HMGB1 showed a slight increase in hydrodynamic diameter (Figure 2B) and a negative shift in the zeta potential of approximately 10 mV (Figure 2C), supporting successful surface modification with HA1 and HMGB1. HPLC analysis further showed that the encapsulation efficiency of diABZI exceeded 90% (Figure 2D).

We further investigated the colloidal stability of HA1@diABZI–HMGB1 under different storage conditions. Throughout a 7‐day observation period, the nanoparticles maintained a stable particle size with negligible aggregation or precipitation in phosphate‐buffered saline (PBS) or PBS containing 10% fetal bovine serum (FBS) at both 4 and 37°C (Figure 2E), indicating favorable in vitro stability.

Drug‐release kinetics were then evaluated under different pH conditions using a dialysis method. HA1@diABZI–HMGB1 showed sustained diABZI release at pH 7.4, with a cumulative release of 70.38% over 120 h. In contrast, release was accelerated under acidic conditions, reaching 81.99% at pH 5.5 over the same period (Figure 2F). This pH‐responsive release profile may help limit nonspecific leakage under physiological conditions while promoting payload release in the acidic TME.

### 2.4 Modulation of Macrophage Phagocytosis and Immune Phenotypes by HA1@diABZI–HMGB1

We investigated whether HA1@diABZI–HMGB1 could enhance phagocytic efficiency and promote M1‐like polarization through macrophage–tumor cell bridging and STING activation. Its immunomodulatory effects were evaluated in an in vitro coculture system comprising bone marrow‐derived macrophages (BMDMs) and ESCC mEC25 cells.

Flow cytometry was used to quantify macrophage phagocytosis of CFSE‐labeled mEC25 cells (Figure 2G). Free diABZI showed only marginal prophagocytic activity, whereas EYLNs–diABZI increased phagocytic efficiency, likely reflecting improved delivery of diABZI. Notably, HA1@diABZI–HMGB1 induced the strongest phagocytic response among all groups. CLSM further confirmed this effect, showing abundant CFSE‐positive tumor‐cell signals within PKH26‐labeled BMDMs after HA1@diABZI–HMGB1 treatment (Figure 2H). To assess the contribution of HMGB1‐associated receptors, BMDMs were pretreated with the TLR4 inhibitor TAK‐242 and/or the RAGE inhibitor FPS–ZM1. Inhibition of either receptor reduced HA1@diABZI–HMGB1‐enhanced phagocytosis, whereas dual inhibition produced a more pronounced decrease (Figure S2A). Consistently, confocal imaging showed reduced tumor‐cell engulfment after receptor blockade (Figure S2B), suggesting that HMGB1 promotes macrophage–tumor cell interaction and phagocytosis at least partly through TLR4/RAGE pathways.

We next examined macrophage polarization. HA1@diABZI–HMGB1 markedly upregulated the costimulatory molecule CD86 on BMDMs (Figure 2I), indicating enhanced M1‐like activation. In interleukin (IL)‐4/IL‐13‐induced M2‐like BMDMs, Notably, HA1@diABZI–HMGB1 treatment was associated with marked immune reprogramming of macrophages, with the M1/M2 phenotype ratio increasing from 0.06 in the control group to 16.16 (Figure 2J). These results support its ability to repolarize macrophages toward an antitumor M1‐like phenotype.

The functional consequence of macrophage reprogramming was further assessed using a Transwell coculture system. mEC25 proliferation was most significantly suppressed in the HA1@diABZI–HMGB1 group (Figure 2K), accompanied by macrophage morphological changes consistent with M1‐like activation, including cell flattening and extension of pseudopodia. Together, these findings support the potential of HA1@diABZI–HMGB1 to modulate macrophage phagocytosis and polarization, and suggest its capacity to remodel the tumor immune microenvironment (TIME). In addition, HA1@diABZI–HMGB1 increased calreticulin exposure and extracellular ATP release in mEC25 cells (Figure S3A,B), indicating induction of ICD‐associated damage‐associated molecular pattern DAMP signals.

### 2.5 HA1@DiABZI–HMGB1 Activates the STING Pathway and Induces the Secretion of Proinflammatory Cytokines

**FIGURE 3:**
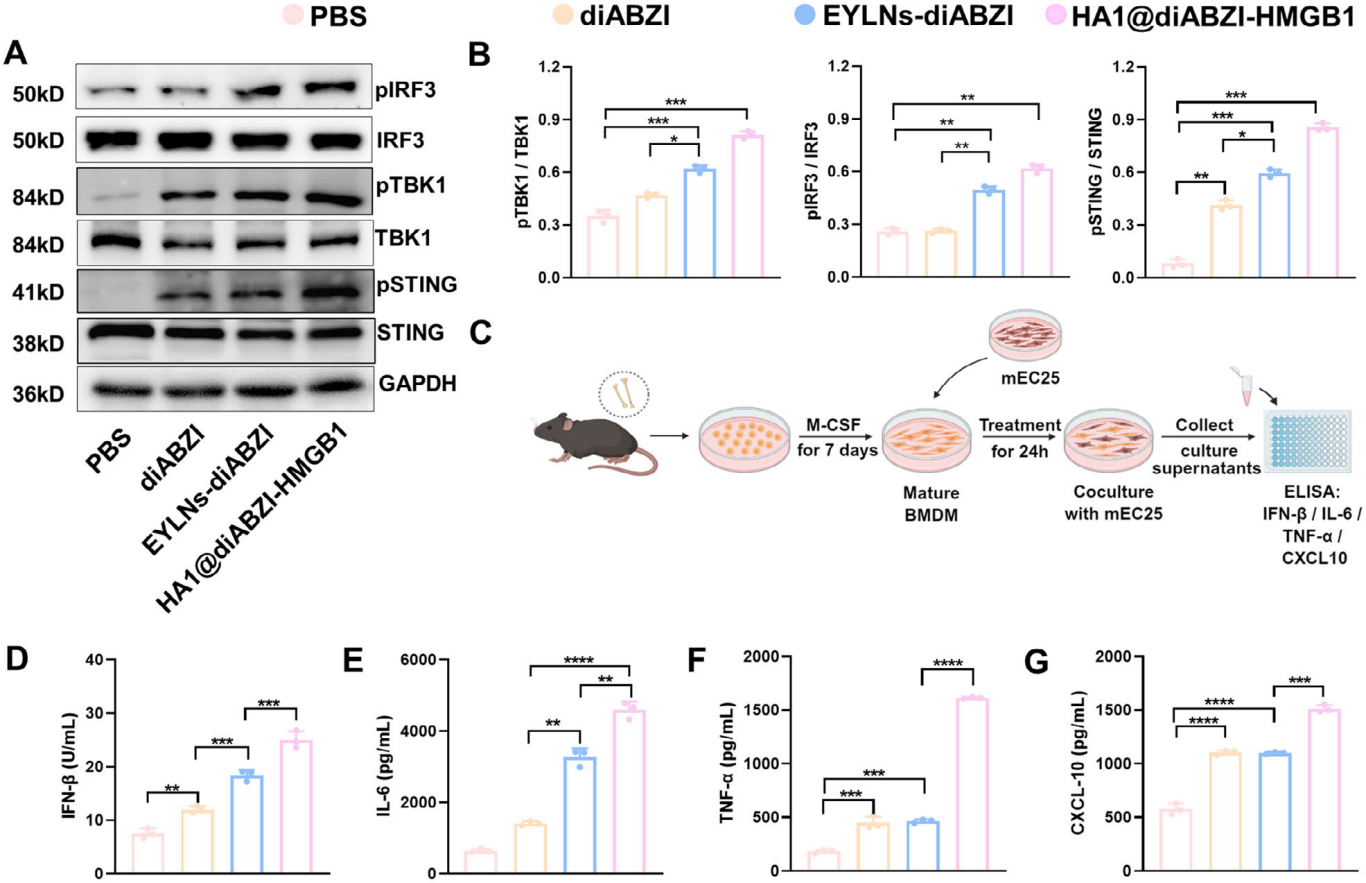
HA1@diABZI–HMGB1 activates the STING signaling pathway in BMDMs and enhances proinflammatory cytokine production. (A) Western blot analysis was conducted to examine STING, TBK1, IRF‐3, and their phosphorylated forms in BMDMs following various treatments. (B) A quantitative assessment of the phosphorylation levels of proteins within the STING signaling pathway was performed. (C) A schematic representation of the experimental design is provided. Primary BMDMs were isolated from murine bone marrow and differentiated using macrophage colony‐stimulating factor (M‐CSF) at a concentration of 20 ng/mL over a period of 7 days. The mature BMDMs were subsequently cocultured with mEC25 tumor cells and exposed to different treatments for 24 h. Culture supernatants were collected and subjected to ELISA to quantify the levels of IFN‐β, IL‐6, TNF‐α, and CXCL10. (D–G) ELISA was utilized to quantify the levels of IFN‐β, IL‐6, TNF‐α, and CXCL10 in the coculture supernatants following the various treatments. All data expressed as mean ± SD (*n* = 3). Statistical significance was calculated through one‐way ANOVA using the Tukey's posttest. *p < 0.05, **p < 0.01, ***p < 0.001, and ****p < 0.0001.

To elucidate the efficacy of different delivery strategies on the macrophage STING signaling pathway, BMDMs were treated with PBS, free diABZI, EYLNs–diABZI, or HA1@diABZI–HMGB1. Western blotting revealed that free diABZI induced only marginal phosphorylation of STING, TBK1, and IRF3, whereas EYLNs–diABZI enhanced phosphorylation signals of these proteins, likely due to improved intracellular delivery. Notably, at equivalent diABZI concentrations, HA1@diABZI–HMGB1 elicited the strongest phosphorylation response in the STING pathway (Figure 3A,B), supporting the advantage of the functionalized nano‐delivery system in promoting STING signaling.

Furthermore, we evaluated inflammatory cytokine production in a BMDM–mEC25 coculture system that mimics the TME (Figure 3C). HA1@diABZI–HMGB1 significantly upregulated the secretion of IFN‐β, IL‐6, TNF‐α, and CXCL10 (Figure 3D–G), indicating robust activation of STING‐associated inflammatory responses. T To functionally validate the role of STING signaling in macrophage repolarization, M2‐like BMDMs were pretreated with the STING inhibitor H‐151 before HA1@diABZI–HMGB1 stimulation. H‐151 markedly suppressed HA1@diABZI–HMGB1‐induced M2‐to‐M1 repolarization, while showing minimal effects on the basal M2‐like phenotype (Figure S4). These results suggest that HA1@diABZI–HMGB1‐mediated macrophage reprogramming is at least partly dependent on STING signaling.

### 2.6 Synergistic Enhancement of In Vivo Biodistribution and Circulatory Stability by HA1 and HMGB1

**FIGURE 4:**
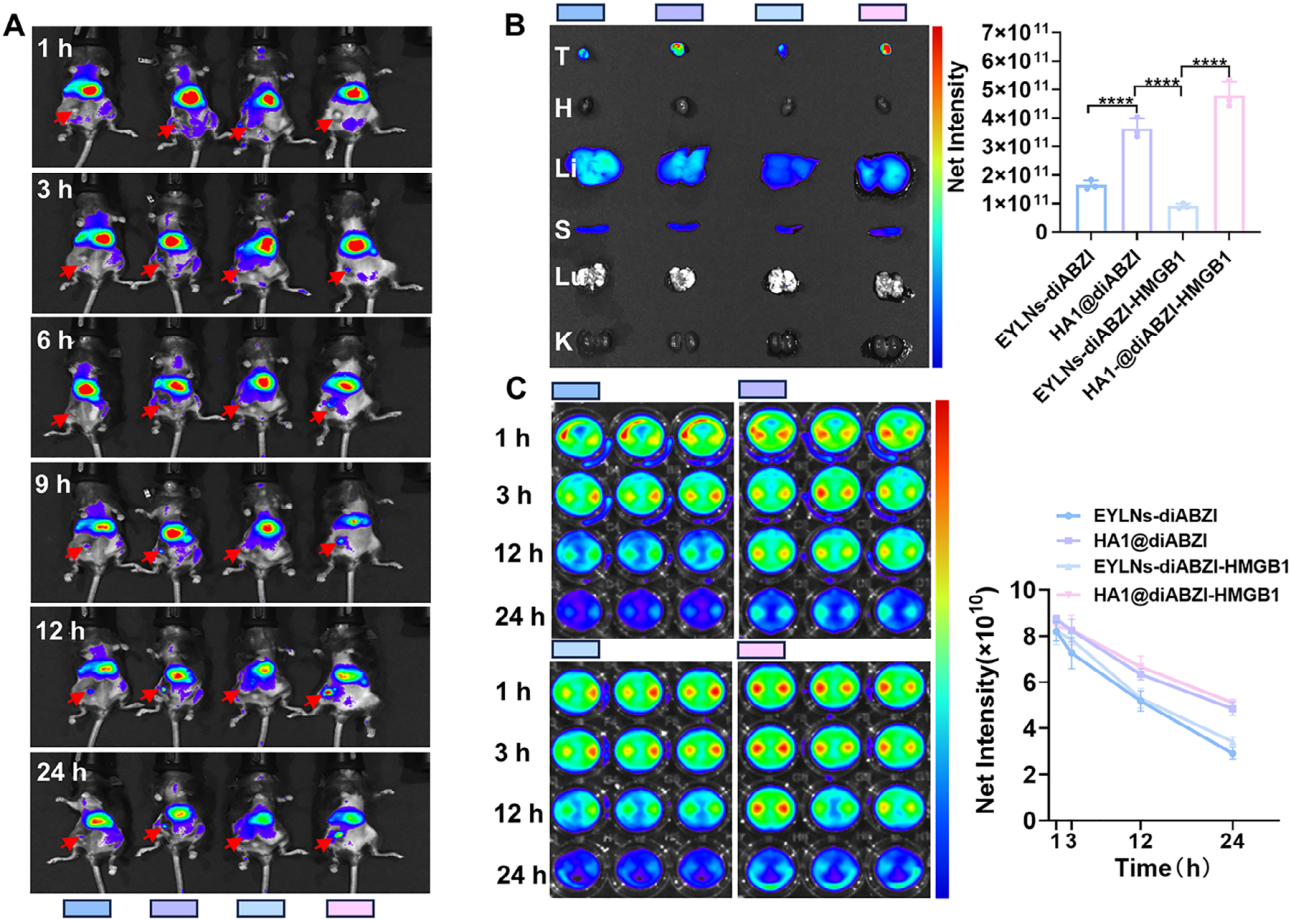
Synergistic enhancement of in vivo biodistribution and circulatory stability by HA1 and HMGB1 (A and B). The in vivo biodistribution of various DiR‐labeled nanoparticles was assessed in mEC25 tumor‐bearing mice at 1, 3, 6, 9, 12, and 24 h following intravenous administration. Tumor regions are marked with red arrows. For ex vivo imaging, the mice were euthanized 24 h postinjection, and both tumors and major organs were harvested for fluorescence imaging. The fluorescence intensity within the tumors was subsequently quantified. (C) The fluorescence signals in the peripheral blood of mEC25 tumor‐bearing mice were measured at different time intervals following the tail vein injection of DiR‐labeled nanoparticles. Data are shown as the mean ± SD (*n* = 3). Statistical significance was calculated through one‐way ANOVA using the Tukey's posttest. ****p < 0.0001.

To evaluate the effect of surface functionalization on in vivo biodistribution, we established an mEC25 subcutaneous tumor‐bearing mouse model and intravenously administered four DiR‐labeled formulations: EYLNs–diABZI, HA1@diABZI, EYLNs‐diABZI–HMGB1, and HA1@diABZI–HMGB1. Real‐time fluorescence imaging showed that HA1@diABZI–HMGB1 exhibited superior tumor‐homing capabilities, with detectable tumor enrichment as early as 3 h postinjection and consistently higher tumor fluorescence than the control formulations from 1 to 24 h (Figure 4A). Ex vivo imaging at 24 h further confirmed enhanced tumor accumulation in HA1‐modified formulations, particularly HA1@diABZI–HMGB1 and HA1@diABZI, compared with nontargeted groups (Figure 4B), supporting the contribution of HA1 to tumor‐associated nanoparticle accumulation.

Peripheral blood fluorescence monitoring further revealed distinct circulation profiles among the formulations. EYLNs–diABZI showed the most rapid fluorescence decay, whereas HA1 or HMGB1 modification prolonged blood retention. Notably, HA1@diABZI–HMGB1 maintained the highest DiR‐derived blood fluorescence at all time points (Figure 4C), suggesting improved circulatory stability. This enhanced circulation profile is likely associated with the surface architecture formed by HA1 and HMGB1. These modifications may optimize surface charge and steric hindrance, thereby reducing nonspecific plasma protein adsorption and uptake by the mononuclear phagocyte system.

In summary, the combined effects of HA1‐mediated tumor targeting and HMGB1‐associated long‐circulation properties may contribute to a favorable “long‐circulation plus tumor‐targeting” in vivo behavior. These findings support the potential of HA1@diABZI–HMGB1 to enhance drug enrichment at the tumor site and provide a basis for subsequent immune activation.

### 2.7 Evaluation of In Vivo Antitumor Efficacy of HA1@diABZI–HMGB1

**FIGURE 5:**
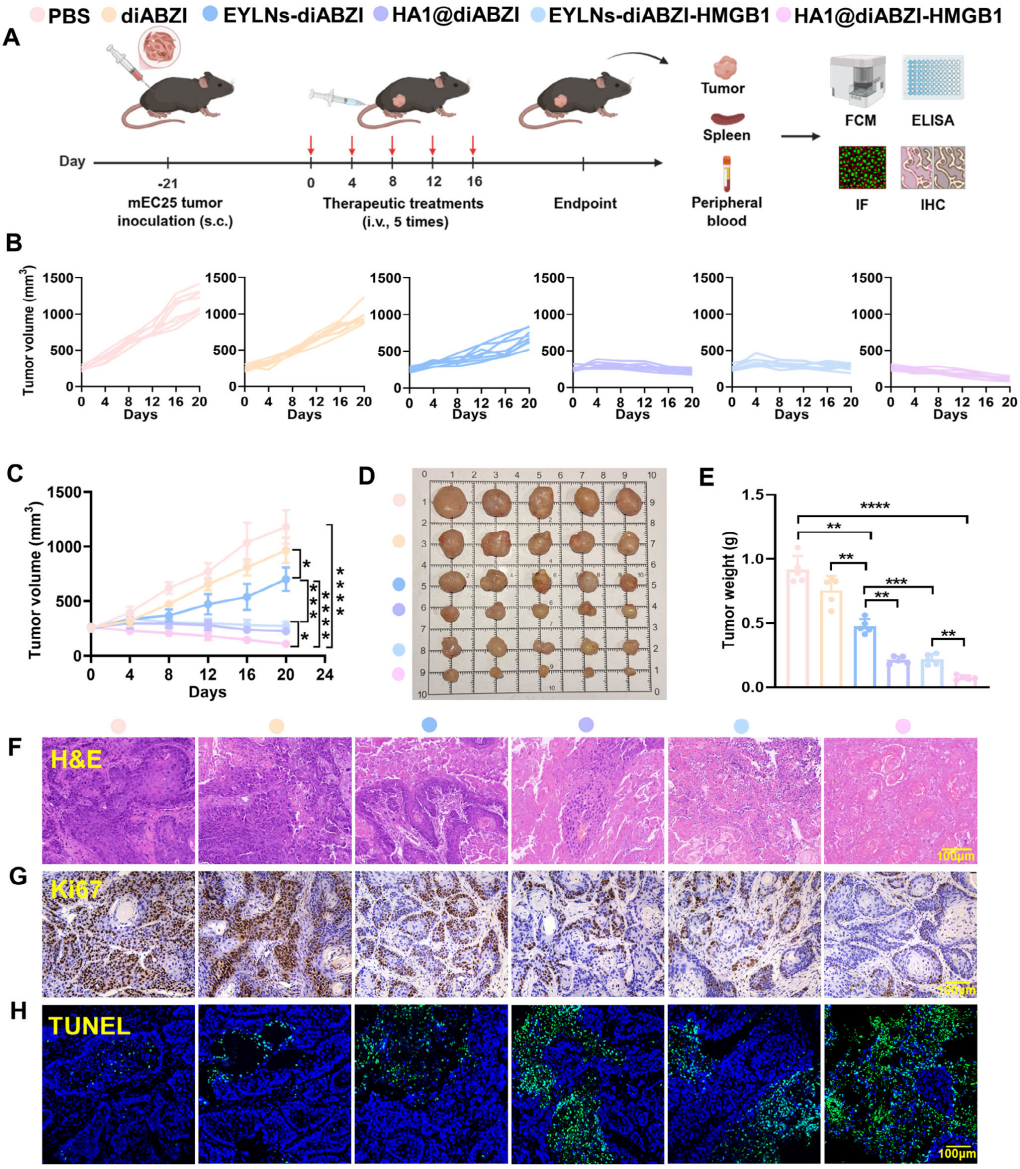
Evaluation of in vivo antitumor efficacy of HA1@diABZI–HMGB1 (A). A schematic representation of the treatment regimen, encompassing PBS, free diABZI, EYLNs–diABZI, HA1@diABZI, EYLNs–diABZI–HMGB1, and HA1@diABZI–HMGB1. (B) Individual tumor growth trajectories of mEC25 tumor‐bearing mice subjected to various treatments. (C) Mean tumor growth trajectories of mEC25 tumor‐bearing mice within each treatment group. Data are shown as the mean ± SD (*n* = 8). Statistical significance was calculated through one‐way ANOVA using the Tukey's posttest. *p < 0.05, ***p < 0.001, and ****p < 0.0001. (D and E) Representative images of excised tumors and corresponding tumor weights across different treatment groups. Data are shown as the mean ± SD (*n* = 5). Statistical significance was calculated through one‐way ANOVA using the Tukey's posttest. **p < 0.01, ***p < 0.001, and ****p < 0.0001. (F–H) Representative images of H&E, Ki67, and TUNEL staining of tumor sections from each treatment group.

We conducted therapeutic evaluations in a C57BL/6 subcutaneous model (mEC25) to systematically assess the antitumor efficacy of the fully functionalized HA1@diABZI–HMGB1 nano‐formulation. To further explore the pharmacodynamic contributions of the distinct functional modules, the experimental design incorporated single modification groups (HA1@diABZI and EYLNs–diABZI–HMGB1) as critical controls. This approach allowed us to assess the potential cooperative effects between HA1‐mediated active targeting and HMGB1‐associated immunomodulation. Upon reaching a tumor volume of 200–300 mm^3^, the mice were randomized into six treatment cohorts (PBS, free diABZI, EYLNs–diABZI, HA1@diABZI, EYLNs–diABZI–HMGB1, and HA1@diABZI–HMGB1) and administered five intravenous injections according to the scheduled protocol (Figure 5A).

Monitoring of tumor growth kinetics indicated that free diABZI and the basic carrier group (EYLNs—iABZI) exhibited only marginal tumor‐suppressive activity. This result suggests that nontargeted administration may have limited the effective accumulation of diABZI at the tumor site. In comparison, groups with single functional modifications (HA1@diABZI or EYLNs‐diABZI–HMGB1) displayed moderate tumor inhibition. Notably, the dual‐functionalized HA1@diABZI–HMGB1 group achieved the strongest suppression of tumor growth among all tested groups (Figure 5B,C). This finding was further supported by ex vivo tumor images and tumor weight analysis at the therapeutic endpoint (Figure 5D, E). These endpoint data were broadly consistent with the tumor volume results and showed significantly lower tumor burden in the HA1@diABZI–HMGB1 group than in the other control groups. Together, these results suggest that the integration of active targeting and immune‐enhancing modules may improve the therapeutic performance of this nano‐formulation.

Subsequent histopathological assessments were consistent with the observed macroscopic efficacy. H&E‐stained sections from the HA1@diABZI–HMGB1 treatment group revealed extensive nuclear pyknosis, fragmentation, and tissue architectural disintegration (Figure 5F), indicating marked tumor cell damage. Furthermore, Ki67 immunohistochemical analysis showed a significantly reduced tumor cell proliferation index in this group (Figure 5G), whereas TUNEL assays showed increased apoptotic signals (Figure 5H). Collectively, these data support the superior antitumor activity of HA1@diABZI–HMGB1, which was associated with reduced tumor cell proliferation and increased apoptosis. The enhanced efficacy of this formulation may be related to the combined effects of HA1‐mediated tumor targeting, HMGB1‐facilitated cellular interaction, and potent STING pathway activation, which together may contribute to a more favorable anti‐TIME.

### 2.8 HA1@DiABZI–HMGB1 Remodels the TIME and Activates Systemic Antitumor Immunity

**FIGURE 6:**
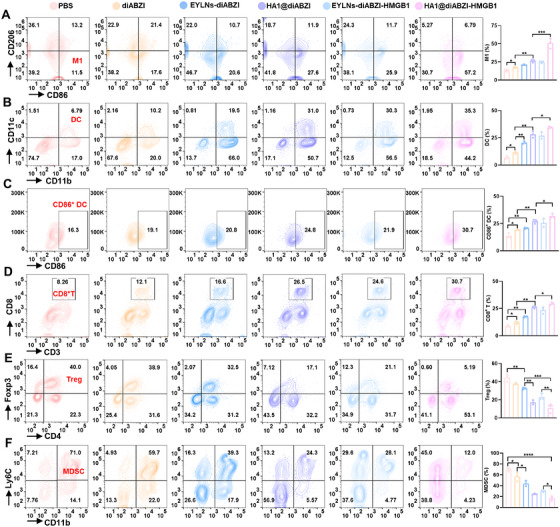
HA1@diABZI–HMGB1 markedly improves the tumor immune microenvironment. (A) Flow cytometric analysis of tumor‐infiltrating M1‐like macrophages (CD45^+^CD11b^+^F4/80^+^CD86^+^CD206^−^). (B and C) Flow cytometric analysis of tumor‐infiltrating dendritic cells DCs (CD45^+^CD11b^+^CD11c^+^) and mature DCs (CD45^+^CD11b^+^CD11c^+^CD86^+^). (D) Flow cytometric analysis of tumor‐infiltrating CD8^+^ T cells (CD45^+^CD3^+^CD8^+^). (E) Flow cytometric analysis of tumor‐infiltrating regulatory T cells Tregs (CD45^+^CD3^+^CD4^+^Foxp3^+^). (F) Flow cytometric analysis of tumor‐infiltrating myeloid‐derived suppressor cells MDSCs (CD45^+^CD11b^+^Ly‐6C^+^). All data are shown as the mean ± SD (*n* = 3). Statistical significance was calculated through one‐way ANOVA using the Tukey's posttest. *p < 0.05, **p < 0.01, ***p < 0.001, and ****p < 0.0001.

To gain mechanistic insights into antitumor immunity mediated by HA1@diABZI–HMGB1, we systematically evaluated its potential to modulate the local tumor TIME and activate the systemic immune system. Flow cytometry analysis demonstrated that treatment with HA1@diABZI–HMGB1 was associated with a significant immunophenotypic shift. The gating strategy for immune cell detection is shown in Figures S5 and S6. In the context of innate immunity, the treatment promoted the polarization of TAMs toward an antitumor M1‐like phenotype, increasing from 11.5% in the PBS group to 57.2% and significantly exceeding the levels observed in the single‐modification groups (Figure 6A). It also markedly increased the infiltration abundance and maturation (CD86^+^) of DCs, suggesting enhanced antigen‐presenting activity (Figure 6B,C). This more proinflammatory microenvironment may further support adaptive immune activation, as indicated by a substantial increase in the proportion of infiltrating effector CD8^+^ T cells (Figure 6D). Meanwhile, HA1@diABZI–HMGB1 reduced key immunosuppressive cell populations within the tumor. Following treatment, regulatory T cells (Tregs) decreased from 40 to 5.19%, and myeloid‐derived suppressor cells (MDSCs) decreased from 71.0 to 12.0% (Figure 6E,F). These findings suggest that the nanosystem may help alleviate the local immunosuppressive state by enhancing immune‐activating cell populations while reducing immunosuppressive components.

**FIGURE 7:**
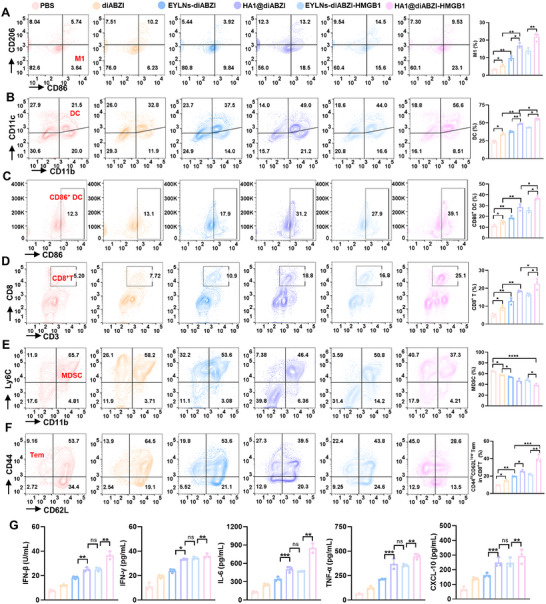
HA1@diABZI–HMGB1 activates systemic antitumor immunity. (A) Flow cytometric analysis of M1‐like macrophages (CD45^+^CD11b^+^F4/80^+^CD86^+^CD206^−^) in the spleen. (B and C) Flow cytometric analysis of splenic dendritic cells DCs (CD45^+^CD11b^+^CD11c^+^) and mature DCs (CD45^+^CD11b^+^CD11c^+^CD86^+^). (D) Flow cytometric analysis of splenic CD8^+^ T cells (CD45^+^CD3^+^CD8^+^). (E) Flow cytometric analysis of splenic myeloid‐derived suppressor cells MDSCs (CD45^+^CD11b^+^Ly‐6C^+^). (F) Flow cytometric analysis of splenic effector memory CD8^+^ T cells Tem (CD45^+^CD3^+^CD8^+^CD44^hi^CD62^Low^). (G) Serum levels of IFN‐β, IL‐6, TNF‐α, and CXCL‐10 in different treatment groups. All data are shown as the mean ± SD (*n* = 3). Statistical significance was calculated through one‐way ANOVA using the Tukey's posttest. *p < 0.05, **p < 0.01, ***p < 0.001, and ****p < 0.0001.

In addition to its effects on the local TIME, HA1@diABZI–HMGB1 was associated with enhanced systemic antitumor immune responses. In the spleen, the proportions of both M1‐type macrophages and dendritic cells (DCs) were significantly upregulated (Figure 7A–C). Meanwhile, the infiltration ratio of effector CD8^+^ T cells was also substantially increased (Figure 7D), Furthermore, the proportion of MDSCs in the spleen was significantly downregulated (Figure 7E). More importantly, the proportion of effector memory T cells (Tem) was significantly elevated (Figure 7F), suggesting the potential induction of immune memory‐like responses. The gating strategy is shown in Figure S3. Serum cytokine analysis further supported these immune changes. IFN‐β, IFN‐γ, IL‐6, TNF‐α, and CXCL‐10 levels in the HA1@diABZI–HMGB1 group were significantly higher than those in all other groups (Figure 7G), indicating the successful activation of a robust systemic proinflammatory and immune response.

Collectively, HA1@diABZI–HMGB1 treatment was associated with a shift of ESCC tumors toward a more immunologically active phenotype by promoting innate and adaptive immune cell activation while reducing immunosuppressive populations. These findings support its potential to modulate the TIME and enhance systemic antitumor immunity, although further studies are needed to determine the durability of this immune remodeling.

### 2.9 HA1@DiABZI–HMGB1 Exhibits an Excellent Biosafety Profile

**FIGURE 8:**
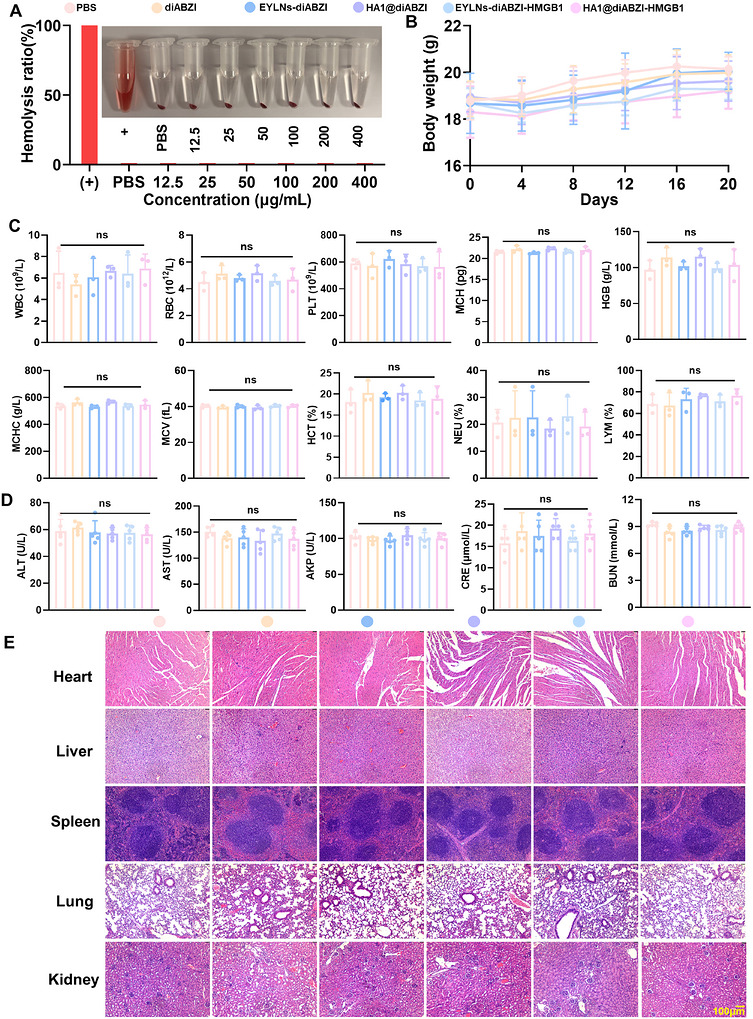
HA1@diABZI–HMGB1 exhibits an excellent biosafety profile (A). Hemolysis assay of HA1@diABZI–HMGB1 across varying concentrations. (B) Variations in body weight observed throughout the treatment period. (C) Hematological analysis conducted on treated mice, encompassing white blood cells (WBC), red blood cells (RBC), platelets (PLT), mean corpuscular hemoglobin (MCH), hemoglobin (HGB), mean corpuscular hemoglobin concentration (MCHC), mean corpuscular volume (MCV), hematocrit (HCT), neutrophils (NEU), and lymphocytes (LYM). (D) Serum biochemical analysis performed on treated mice, with liver function indicators including alanine aminotransferase (ALT), aspartate aminotransferase (AST), and alkaline phosphatase (AKP), and kidney function indicators comprising creatinine (CRE) and blood urea nitrogen (BUN). (E) Histopathological examinations using hematoxylin and eosin (HE) staining of the heart, liver, spleen, lung, and kidney, with a scale bar of 100 µm. For hemolysis assay and hematological analysis, data expressed as mean ± SD (*n* = 3); for body weight variation curves, data expressed as mean ± SD (*n* = 8); for serum biochemical analysis, data expressed as mean ± SD (*n* = 5). Statistical significance between different groups was obtained by one‐way ANOVA using the Tukey's posttest.

Superior biocompatibility is a prerequisite for clinical translation of nanomedicines. In this study, we evaluated the biosafety of HA1@diABZI–HMGB1 in terms of hemocompatibility, systemic toxicity, and histopathology. In vitro hemolysis assays demonstrated that the nano‐formulation showed favorable hemocompatibility. Even at a high incubation concentration of 400 µg/mL, the hemolysis rate was maintained below 2% (Figure 8A). This value is below the commonly accepted safety threshold, supporting the feasibility of intravenous administration. Notably, throughout the therapeutic monitoring period, body weights across all groups showed a stable growth trend (Figure 8B), with no overt systemic toxicity observed. These results suggest a favorable biocompatibility and safety profile of the nanocarrier system under the tested conditions.

Subsequently, we conducted an in vivo toxicity assessment in mice following completion of the therapeutic cycle. Complete blood count (CBC) analysis (Figure 8C) and serum biochemical analysis (Figure 8D) revealed that hematological parameters and indicators of liver and kidney function in the HA1@diABZI–HMGB1 treatment group were within normal physiological ranges. Furthermore, no statistically significant differences were observed compared with the PBS control group, suggesting that the treatment did not induce apparent myelosuppression or systemic biochemical disorders. Additionally, histopathological examination by H&E staining of major organs, including the heart, liver, spleen, lungs, and kidneys, revealed no discernible tissue damage, inflammatory infiltration, or necrotic lesions (Figure 8E). Collectively, these results support the favorable biosafety profile of HA1@diABZI–HMGB1 and suggest that it can exert antitumor effects without causing apparent systemic toxicity in the current experimental setting, thereby supporting its potential for further preclinical evaluation.

## 3 Discussion

Recently, the paradigm of tumor immunotherapy has gradually shifted from a singular focus on adaptive immunity to a broader appreciation of innate immunity [21]. The cGAS–STING signaling pathway serves as a key molecular link between innate and adaptive immunity, has been implicated in antitumor immune surveillance, and represents a potential target for modulating immunosuppressive TMEs [22, 23, 24]. Activation of this pathway triggers a IFN‐I response, supporting DC antigen presentation and effector T‐cell recruitment [25, 26]. In addition, STING agonists can reshape macrophage phenotypes, reversing the immunosuppressive functions of M2‐like TAMs [27]. However, the clinical translation of small‐molecule STING agonists, such as diABZI, remains limited by systemic toxicity, insufficient tumor accumulation, and inefficient intracellular delivery [28, 29]. For “immunologically cold” malignancies such as ESCC, monotherapy relying solely on STING activation may be insufficient, due to multifaceted immune evasion mechanisms and limited effector T‐cell infiltration [30, 31]. Therefore, intelligent nanosystems integrating targeted delivery with coordinated “phagocytosis–polarization” regulation and complementary immune signaling may help overcome barriers in ESCC immunotherapy.

This study innovatively engineered a multifunctional nanoplatform, HA1@diABZI–HMGB1, to modulate immune tolerance in ESCC through a potential dual mechanism involving “physical bridging” and “biochemical activation.” Unlike previous studies that delivered single payloads or primarily blocked signaling pathways [32, 33], our design reflects an integration of precision medicine and engineering principles. By leveraging the HA1aptamer to target membrane proteins expressed in ESCC, our results suggested enhanced tumor‐site accumulation and improved intratumoral distribution of nanoparticles, which may reduce off‐target toxicity and is consistent with “active targeting nanomedicine” advocated by Li et al. [34]. HMGB1 is introduced as a multifunctional component. Beyond its “eat me” signal, HMGB1 may function as a molecular bridge by interacting with TLR4 and RAGE receptors, thereby facilitating spatial proximity between macrophages and tumor cells [35, 36]. Our findings suggest that the HA1‐ and HMGB1‐mediated bridging effect enhances macrophage–tumor cell interactions and may partially relieve physical constraints that limit phagocytosis in solid tumors. This strategy shares a conceptual parallel with bispecific antibodies to bridge T cells and tumor cells, whereas our platform focuses on macrophage‐mediated innate immune responses to initiate antitumor immune activation earlier in the immune cycle [37].

Based on macrophage–tumor cell proximity and enhanced phagocytosis, controlled release of the second‐generation STING agonist diABZI from the nanoparticle core may further amplify antitumor immune response. Phagocytosis may facilitate exposure or transfer of tumor‐derived DNA into the macrophage cytosol, priming cGAS activation. Concurrently, intracellular release of diABZI is expected to activate STING signaling, as supported by increased downstream TBK1/IRF3 signaling observed in our study. Synergistic stimulation by “exogenous drugs” and “endogenous DNA” results in a robust IFN‐I positive feedback loop [38, 39]. Our data further suggest a shift in the immune landscape: TAMs displayed a tendency to repolarize from an M2‐like phenotype toward an M1‐like phenotype, accompanied by increased IL‐12, TNF‐α, and CXCL10, which may support DC recruitment and activation 40]. Mature DCs may bridge innate immune activation with adaptive immune responses. In vivo results also supported a more immunologically active TME, with increased CD8+ and CD4+ T‐cell infiltration and reduced suppressive populations, including Tregs and MDSCs. This comprehensive remodeling curbs primary tumor growth and lays a foundation for long‐term immunological memory and inhibition of distant metastatic potential, which is difficult to achieve with monotherapies [41].

Despite the encouraging efficacy observed in preclinical models, several limitations should be acknowledged as this strategy moves toward clinical translation. First, the long‐term fate of nanoparticles and their interactions with heterogeneous macrophage subsets, such as tissue‐resident and monocyte‐derived macrophages, remain to be clarified through lineage tracing studies [42]. Second, although this study focused on the STING/TBK1/IRF3 signaling axis, the potential contribution of HMGB1‐mediated NF‐κB activation via the TLR4–MyD88 pathway cannot be overlooked. Crosstalk between these pathways requires further investigation using conditional gene knockout models [43, 44]. Furthermore, given the high genomic heterogeneity of ESCC, evaluating whether this strategy is broadly applicable across TP53‐ or NOTCH1‐defined molecular subtypes, or in models with varying PD‐L1 expression levels, will be important for patient stratification [45, 46]. Finally, systematic pharmacokinetic analyses and long‐term safety assessments, autoimmune risks, remain essential thresholds before clinical application.

In summary, this study developed a nano‐immunomodulator, HA1@diABZI–HMGB1, integrating “targeted anchoring, physical bridging, and immune activation.” By employing a “bridge‐and‐activate” strategy, this platform may alleviate macrophage phagocytic constraints in solid tumors and support multidimensional modulation of the ESCC immune microenvironment, potentially through coordinated activation of the STING/TBK1/IRF3 pathway and enhancement of macrophage‐mediated immune responses. These findings suggest that HA1@diABZI–HMGB1 represents a promising combination strategy for addressing immune resistance in ESCC and may provide a useful framework for coordinating innate and adaptive immunity in solid tumor treatment.

## 4 Materials And Methods

### 4.1 Materials

The following materials were procured for this study: diABZI STING agonist‐1, DSPE–PEG 2000, DSPE–PEG–azide, macrophage colony‐stimulating factor (M‐CSF), IL‐4, IL‐13, PKH 26, and CFSE from MedChemExpress; branched PEI (Mw ∼ 25,000) and 4,6‐diamidino‐2‐phenylindole (DAPI) from Sigma–Aldrich; DMEM medium and FBS from Gibco; an anti‐Ki67 antibody from Abcam; and a one‐step TUNEL in situ apoptosis kit from Elabscience. Additionally, commercial reagent kits for the determination of alanine transaminase, aspartate transaminase, alkaline phosphatase, creatinine, and blood urea nitrogen were supplied by Nanjing JianCheng Bioengineering Institute (Nanjing, China). Furthermore, a mouse precoated ELISA kit was obtained from Dakewe Biotech Co. Ltd (Shenzhen, China), and Mouse ELISA Kits for IFN‐β, IFN‐γ, TNF‐α, and CXCL‐10 were acquired from Neobioscience.

### 4.2 Animals

Female C57BL/6 mice were procured from GemPharmatech Co., Ltd. (Nanjing, China). All animal experiments were conducted with the approval of the Animal Care and Use Committee of The Affiliated Huai'an No. 1 People's Hospital of Nanjing Medical University (Approval No. DW‐P‐2024‐008‐01).

### 4.3 Preparation of HA1@diABZI–HMGB1 Nanoparticle

Initially, polar lipids were extracted from egg yolks utilizing a kit specifically designed for the separation of polar and neutral lipids. Subsequently, EYLNs were prepared in accordance with established protocols from prior research. For the formulation of EYLNs–diABZI, the extracted lipids (3 mg) and diABZI (22.15 µg) were dissolved in chloroform, resulting in a clear solution achieved through ultrasonic treatment. This solution was then subjected to drying in a forced‐draft oven to form a thin film complex. The resultant complex was hydrated with 200 µL of PBS and underwent sonication for 20 min. To eliminate any unencapsulated diABZI, the mixture was centrifuged at 4°C and 10,000 rpm for 20 min.

To synthesize HA1@diABZI–HMGB1, EYLNs–diABZI were initially combined with PEI (82.5 µg) and subjected to agitation in an ice bath for 20 min. Subsequently, HA1 (3 nmol) was introduced, followed by an additional 20 min of agitation. The resultant mixture was then sonicated. Thereafter, 1 mol% DSPE–PEG2000–Azide and 3 mol% DSPE–PEG2000 were incorporated, and the reaction was allowed to proceed overnight at room temperature to form azide‐functionalized liposomes. Concurrently, HMGB1 was modified using DBCO–PEG4–NHS to yield DBCO–HMGB1. For the preparation of HA1@diABZI–HMGB1, EYLNs–diABZI were mixed with PEI (82.5 µg) and stirred for 20 min in an ice bath. HA1 (3 nmol) was then added, followed by another 20 min of stirring. The mixture underwent ultrasonic treatment, after which 1% DSPE–PEG2000–Azide and 3% DSPE–PEG2000 were added, allowing the reaction to proceed overnight at room temperature to construct azide‐functionalized liposomes. HMGB1 was modified with DBCO–PEG4–NHS to produce DBCO–HMGB1. The two components were then combined and reacted at 4°C for 12 h to yield the final product, HA1@diABZI–HMGB1. Unbound substances were removed via centrifugation at 4°C, 10,000 rpm for 20 min. The preparation of HA1@diABZI and EYLNs–diABZI–HMGB1 followed analogous procedures.

### 4.4 BMDM Extraction

BMDMs were isolated from 6‐ to 8‐week‐old female C57BL/6 mice and cultured in complete DMEM medium supplemented with M‐CSF at a concentration of 20 ng/mL (Sangon Biotech) at 37°C in an atmosphere containing 5% (v/v) CO_2_. After a 7‐day incubation period, adherent cells were identified as BMDMs and harvested for subsequent experimental procedures.

### 4.5 In Vitro Phagocytosis Assay

For the in vitro phagocytosis assay, BMDM cells were seeded at a density of 4 × 10^5^ cells per well in a six‐well plate and allowed to adhere overnight. Subsequently, 1 × 10^5^ CFSE‐labeled mEC25 cells were introduced and cocultured for 18 h at 37°C. The BMDMs were then stained with BV785 anti‐CD11b (BioLegend; 101243) to identify the cell population. Phagocytosis was quantified as the percentage of CFSE^+^CD11b^+^ cells within the total CD11b^+^ BMDM population. For confocal microscopy analysis, BMDM cells were labeled with PKH 26.

### 4.6 In Vitro BMDM Phenotype Analysis

Mature BMDMs were exposed to PBS, diABZI (1 µM), EYLNs–diABZI (1 µM), and HA1@diABZI–HMGB1 (1 µM) for a duration of 24 h. Subsequently, samples were collected for the assessment of macrophage markers via flow cytometry, specifically targeting CD11b, F4/80, and CD86. To evaluate the capacity of HA1@diABZI–HMGB1 to reverse the M2 phenotype in BMDMs, the cells were initially stimulated with IL‐4 (20 ng/mL) and IL‐13 (20 ng/mL) for 24 h. Following this, various treatments were administered for an additional 24 h to facilitate phenotypic analysis through flow cytometry, focusing on CD11b, F4/80, CD86, and CD206 markers. The antibodies employed in this study included FITC anti‐CD11b (Invitrogen; 11‐0112‐82), BV785 anti‐F4/80 (BioLegend; 123141), eFluor 450 anti‐CD86 (Invitrogen; 48‐0862‐82), and BV650 anti‐CD206 (BioLegend; 141723).

### 4.7 In Vitro Transwell Coculture

To assess the tumoricidal potential of macrophages treated with HA1@diABZI, we developed a transwell coculture system. In this setup, tumor cells were seeded in the upper chamber, while macrophages were placed in the lower chamber. Following a 24‐h treatment period, the tumor cells in the upper chamber were stained with crystal violet to evaluate cell proliferation. Concurrently, the morphology of the macrophages in the lower chamber was examined microscopically.

### 4.8 Western Blotting

BMDMs were exposed to various formulations for a duration of 12 h. Subsequently, the cells were harvested and lysed on ice using RIPA buffer, which included 1 mM PMSF, 1 mM protease inhibitor, and 1 mM phosphatase inhibitor. The resulting lysate was subjected to centrifugation at 12,000 rpm for 15 min at 4°C to isolate the supernatant containing the total cellular proteins. Protein concentration was quantified utilizing the BCA protein assay (Thermo Fisher Scientific). Proteins were then denatured in a fivefold concentrated solution and resolved via SDS‐PAGE at 95°C. The separated proteins were transferred onto a polyvinylidene difluoride (PVDF) membrane (Millipore, USA). The membrane was blocked with 5% BSA at room temperature for 2 h. Primary antibodies were incubated with the membrane overnight at 4°C. Following washes with TBST, the membrane was incubated with a horseradish peroxidase‐conjugated secondary antibody at room temperature for 1 h. The relative expression levels of the proteins were detected using ECL reagent (Zeta Life, USA).

### 4.9 Cytokine Detection

The cell coculture supernatant was subjected to centrifugation at 10,000 rpm for 10 min, after which the supernatant was collected for assay. Serum samples were obtained from blood following two rounds of centrifugation at 5000 rpm at 4°C. All samples were analyzed according to the manufacturer's instructions for the ELISA kit.

### 4.10 In Vivo Therapeutic Efficacy

To investigate the therapeutic effects of HA1@diABZI–HMGB1 on tumor progression, 6‐week‐old C57BL/6 mice were utilized to develop tumor‐bearing mouse models. A 50 µL single‐cell suspension of mEC25 cells (5 × 10^6^) combined with 50 µL of Matrigel was subcutaneously injected into the mice. Upon the tumors reaching a volume of 200–300 mm^3^, the mice were randomly allocated to the following treatment groups: PBS (0.2 mL), diABZI (2.5 mg/kg), EYLNs–diABZI (2.5 mg/kg), HA1@diABZI (2.5 mg/kg), EYLNs–diABZI–HMGB1 (2.5 mg/kg), and HA1@diABZI–HMGB1 (2.5 mg/kg). The treatments were administered intravenously to the mice on five separate occasions. Tumor volume (mm^3^) was assessed every 3 days and calculated using the formula: tumor volume (mm^3^) = length × width × width/2.

### 4.11 In Vivo Toxicity Evaluation of HA1@diABZI–HMGB1

To evaluate the systemic toxicity induced by the treatment, the body weight of tumor‐bearing mice was monitored every 3 days to detect any signs of adverse reactions. Upon completion of the treatment protocol, the mice were euthanized humanely, and key organs, including the heart, liver, spleen, lungs, and kidneys, were meticulously extracted for comprehensive histopathological examination. These organs were promptly fixed in a 4% paraformaldehyde solution to preserve tissue integrity. Following fixation, the tissues were embedded in paraffin, sectioned uniformly at a thickness of 5 µm, and stained with H&E for microscopic analysis to assess any histological alterations indicative of toxic effects. The stained sections were imaged using a microscope to provide visual documentation of tissue morphology. In addition to histopathological analysis, blood biochemical parameters were assessed using standard diagnostic kits in accordance with the manufacturer's specifications, and a comprehensive hematological analysis was conducted using the Automated Hematology Analyzer BC‐5100 (Mindray) to further evaluate systemic effects.

### 4.12 Statistical Analysis

Data were processed and visualized utilizing GraphPad Prism (version 8.0). All data are presented as mean ± standard deviation (SD). and error bars represent SD unless otherwise stated. The number of biological replicates or animals used in each experiment is indicated in the corresponding figure legends. Biological replicates were defined as independent animals, independently prepared nanoparticle batches, or independently performed cell culture experiments. Technical replicates were defined as repeated measurements or repeated assays performed on the same biological sample. For comparisons between two groups, an unpaired two‐tailed Student's *t*‐test was used. For comparisons among more than two groups, one‐way analysis of variance (ANOVA) was performed followed by Tukey's multiple‐comparisons test. For datasets involving two independent variables, such as treatment group and time, two‐way ANOVA was performed followed by Tukey's or Sidak's multiple‐comparisons test, as appropriate. For longitudinal datasets measured repeatedly in the same animals or samples, repeated‐measures two‐way ANOVA was used where appropriate. A *p* value of less than 0.05 was considered statistically significant. During the data analysis phase of this study, a blind analysis was performed by research team members who were not involved in the evaluation of the experimental implementation. Prior to analysis, the grouping variables were encoded, ensuring that the analysts remained unaware of the correspondence between groupings and treatments until the completion of the primary analysis. This methodology effectively mitigated the potential for analytical bias.

Detailed experimental procedures are provided in the Materials and Methods section of the Supplementary Information, including “Cell lines,” “ssDNA Library and Primers,” “Cell‐SELEX Process,” “Binding Analysis of HA1,” “Modification of HA1 and HMGB1,” “Characterization of HA1@diABZI‐HMGB1,” “Antitumor Immunity Effect In Vivo,” “In Vivo Imaging,” “Detection of peripheral blood circulation behavior,” “H&E, Ki67 and TUNEL assays,” and “Hemolysis test.”

## Author Contributions

Conceptualization: L.Z., C.L., and Q.W. Methodology: C.H., D.X., C.S., X.T., Y.Z., C.W., and F.X. Investigation: C.H., D.X., C.S., X.T., Y.Z., C.W., F.X., L.Z., C.L., and Q.W. Writing – original draft: C.L. and Q.W. Writing – review and editing: C.L. and Q.W. Funding acquisition: C.L. and Q.W. Resources: F.X. and C.H. Supervision: L.Z., C.L., and Q.W. All authors have read and approved the final manuscript.

## Ethics Approval

All animal experiments were conducted with the approval of the Animal Care and Use Committee of The Affiliated Huai’an No.1 People’s Hospital of Nanjing Medical University (Approval No. DW‐P‐2024‐008‐01)

## Funding

This work was supported by the Jiangsu Provincial Key Research and Development Program (BE2023744), National Natural Science Foundation of China (82272683, 82573633), Northern Jiangsu Clinical Medicine Research Institute's 2024 Projects (HAKY202400403, HAKY202400404), the Natural Science Foundation of Jiangsu Province (BK20231235), the Jiangsu Provincial Medical Key Discipline Cultivation Unit (JSDW202233);the Technology Innovation Team Project of The Affiliated Huaian No. 1 People's Hospital, Nanjing Medical University (YCT202307), and Jiangsu Provincial Graduate Research Innovation Program (KYCX24‐2028).

## Conflicts of Interest

The authors declare no conflicts of interest.

## Supporting information




**Supporting Information**: Mco270828‐sup‐0001‐SupMat.docx

## Data Availability

The authors have nothing to report.
