# Supplementary material for: An Engineered Nano‐Bridging Strategy Remodels the Immune Microenvironment of Esophageal Squamous Cell Carcinoma via STING Pathway Activation
Source: MedComm (2020). 2026 Jun 13;7(6):e70828. doi: 10.1002/mco2.70828 (PMC13263789; doi:10.1002/mco2.70828)
Supplement: Supplementary file 1 — Supporting Information: Mco270828‐sup‐0001‐SupMat.docx [file MCO2-7-e70828-s001.docx]

**An engineered nano-bridging strategy remodels the immune microenvironment of esophageal squamous cell carcinoma via STING pathway activation**

Chaofan Huang^1,^**^#^**, Xin Tang^1,#^, Yixiao Zhao^1,#^, Dafu Xu^2,#^, Congyong Sun^1^,Chenlong Wang^1^, Fei Xie^1^, Fangyi Xu^1^, Chao Luo^1*^, Li Zhang^1*^, Qilong Wang^1, *^

^1^Department of Central Laboratory, The Affiliated Huaian No.1 People's Hospital of Nanjing Medical University, Northern

Jiangsu Institute of Clinical Medicine, Nanjing Medical University, Huai'an, 223300, Jiangsu Province, China.

^2^ Department of Thoracic Surgery, The Affiliated Huaian No. 1 People's Hospital of Nanjing Medical University, Huaian,

223300, China.

^#^These authors contributed equally

^*^Correspondence: Dr. Chao Luo, Dr. Li Zhang and Dr. Qilong Wang,Tel: +86-15861710352;

Email: hayylch@njmu.edu.cn, zhangl@njmu.edu.cn, qlwang@njmu.edu.cn.

**Materials AND METHODS**

**Cell lines**

The mouse esophageal squamous cell carcinoma cell line, mEC25, was generously provided by professor Li Fu from Shenzhen University. The 4T1, CT26, and MC38 cell lines were maintained in our laboratory and authenticated by the China Center for Type Culture Collection (CCTCC). All cell lines were cultured in Dulbecco's Modified Eagle Medium (DMEM) supplemented with 10% FBS and antibiotics, at 37°C in a humidified atmosphere containing 5% CO_2_.

**ssDNA Library and Primers**

The single-stranded DNA (ssDNA) library utilized in this study, with the sequence 5’-AGCCTAAGCCTGTCCAGGAATCG -‘40N’-ATGGCTTAGTGGCACGATTAGGTC-3’ and consisting of 87 nucleotides, includes two primer regions located at the 5’ and 3’ ends for PCR amplification. Additionally, it features a 40-nucleotide random region synthesized with equimolar incorporation of adenine (A), thymine (T), cytosine (C), and guanine (G). For library preparation, the forward primer was modified with a carboxylfluorescein (FAM) label at its 5’ end, designated as 5’-FAM-AGCCTAAGCCTGTCCAGGAATCG-3’, to facilitate binding analysis via flow cytometry (B6-plus, BD Bioscience). The reverse primer was conjugated with biotin at the 5’ end to enable the separation of PCR products using streptavidin-coated sepharose beads, indicated as 5’-Biotin-GACCTAATCGTGCCACTAAGCCAT-3’. The entire ssDNA library and primers were synthesized and purified by Sangon Biotech.

**Cell-SELEX Process**

For the purpose of positive selection, mEC25 cells were incubated with the ssDNA library in a binding buffer composed of 4.5 g/L glucose, 1M MgCl2, 1 mg/mL BSA, and 0.1 mg/mL yeast tRNA. This incubation was conducted on a rotary shaker at 4 °C for a duration of 60 minutes. Following incubation, the cells underwent three washes with a washing buffer containing 4.5 g/L glucose and 5 mM MgCl2 to eliminate unbound sequences. The cells were then subjected to heating at 95 °C for 10 minutes to elute the ssDNA bound to the cell surface. Subsequent centrifugation at 15,000 rpm was performed to remove cell debris. The eluted ssDNA underwent PCR amplification, with conditions set at an initial denaturation for 3 minutes at 95 °C, followed by 35 cycles of 15 seconds at 95 °C, 20 seconds at 68 °C, and 90 seconds at 72 °C, concluding with a final extension of 10 minutes at 72 °C, to enrich the selected pool. The PCR products were then incubated with streptavidin-modified sepharose beads for 30 minutes at room temperature. Denaturation was achieved using 200 mM NaOH for 10 minutes, followed by centrifugation to facilitate separation. The resultant ssDNA library was desalted, quantified, and dried in preparation for the subsequent round of selection. In the thirteenth round of selection, negative selection was implemented to eliminate nonspecifically bound sequences. ssDNA library was initially incubated with MEEC at 4°C for a duration of 30 minutes, following which the unbound DNA present in the supernatant was collected for subsequent rounds of positive selection. During the subsequent selection process, the screening conditions were progressively intensified to improve both specificity and affinity. Specifically, the incubation period for negative selection was extended from 30 to 60 minutes, and the number of washing cycles was increased from three to five. Conversely, the incubation time for positive selection was reduced from 60 to 30 minutes. After completing 20 rounds of selection, the ssDNA was amplified and subcloned into the pUC19 vector within Escherichia coli DH5α cells. The selected clones (HA1) were then sequenced, and their secondary structures were analyzed using the Mfold software.

**Binding Analysis of HA1**

Flow cytometry assays were employed to assess library enhancement. Specifically, ssDNA and the selected aptamers, HA1, labeled with FAM at a concentration of 250 nM, were incubated with mEC25 cells in a binding buffer at 4 °C for 60 minutes. Following three washing steps, the cells were resuspended in 400 μL of binding buffer and analyzed via flow cytometry using the FITC channel. A random DNA sequence was utilized as a control. To ascertain the equilibrium dissociation constants (K_d_) of HA1, varying concentrations (0, 50, 100, 150, 200, 250, 300, and 350 nM) of Cy5-labeled HA1 were incubated with 1×10^5^ mEC25 cells at 4 °C for 60 minutes, and the fluorescence signal was subsequently analyzed by flow cytometry. The Kd value was determined using GraphPad Prism 8 software, applying the one-site saturation equation: Y = B_max_ X/(K_d_ + X).

To evaluate the binding specificity of the identified aptamers, experiments were conducted using both mouse ESCC cell lines (mEC25) and various other tumor cell lines (4T1, CT26, MC38). The cells were incubated with 250 nM of Cy5-labeled HA1 at 4 °C for 60 minutes. Subsequently, the cells underwent washing, fixation with 4% PFA for 20 minutes, and nuclear counterstaining with DAPI at a concentration of 10 μg/mL for 15 minutes. Fluorescence images were acquired using laser scanning confocal microscopy (LSCM) (Nikon, Japan).

**Modification of HA1 and HMGB1**

To evaluate the binding efficiency of HA1 to nanoparticles, various samples, including free HA1, EYLNs-diABZI, free HA1 with EYLNs-diABZI, and HA1@diABZI, were analyzed using a 3% agarose gel electrophoresis. The resulting DNA ladders were visualized and documented utilizing a Bioimage system (UVP, GelDoc-It2 Imager). In order to assess the binding efficiency of HMGB1 to nanoparticles, the samples underwent a 10-minute denaturation process at 95°C in a medium containing a protein loading buffer at a 1:4 ratio, as supplied by Epizyme Biotech. The samples were then subjected to separation via sodium dodecyl sulfate-polyacrylamide gel electrophoresis (SDS-PAGE). Following this, silver staining was conducted using a fast silver staining kit (Beyotime), and the images were subsequently captured for analysis.

**Characterization of HA1@diABZI-HMGB1**

The characterization of nano-delivery systems encompassed the evaluation of parameters such as entrapment efficiency, drug loading, morphology, particle size, zeta potential, and storage stability of HA1@diABZI-HMGB1. To assess the entrapment efficiency (EE%), non-encapsulated diABZI was isolated using a cellulose nitrate membrane. Briefly, 200 μL of HA1@diABZI-HMGB1 was placed in a 0.5 mL ultrafiltration tube (MWCO, 100,000 Da, Millipore), then centrifuged at 4 °C, 10000 rpm for 10 min. The quantities of diABZI in the filtrate were regarded as the non-encapsulated drug content. Concurrently, 200 μL of HA1@diABZI-HMGB1 was ruptured by acetonitrile, and subjected to ultrasonic treatment to liberate diABZI, which was considered the total content of diABZI. Then, the concentration of free diABZI (W_non-encapsulated_) and the initial total diABZI (W_total_) were determined using high-performance liquid chromatography (HPLC) method. The EE% was calculated using the formula: EE (%) = [(W_total_ – W_non-encapsulated_) / W_total_] × 100%. All assays were conducted in triplicate.

Quantification of diABZI was performed utilizing high-performance liquid chromatography (HPLC) with an Agilent 1260 liquid chromatography system (Agilent, USA) and a Waters Symmetry C18 column (4.6 × 150 mm, 3.5 μm, Waters, Milford, MA, USA) maintained at 25 °C. The diode array detector (DAD) wavelength was set at 240 nm, and the mobile phase comprised acetonitrile and 0.02% trifluoroacetic acid, with a flow rate of 0.6 mL/min. The standard curve of diABZI was *Y* = 38.494 × *C* + 1.7799 (linear range: 1–100 μg/mL, *R*^2^ = 0.9999), where *Y* refers to the peak area, and *C* represents the diABZI concentration. The analytical method was validated for precision, accuracy, and sensitivity prior to sample analysis. All assays were conducted in triplicate.

For the analysis of particle size and zeta potential, an appropriate quantity of the prepared liposomes was diluted with PBS and subjected to laser diffraction and zeta potential measurements using a dynamic light scattering (DLS) instrument (PSS Nicomp 380 Z3000). The samples were placed in cuvettes and analyzed at a 90° angle and a temperature of 25 °C. Measurements of particle size, distribution, and zeta potential were conducted in triplicate. For morphological studies, the negative-staining technique was utilized. A droplet of the diluted liposome suspension was applied to a specialized copper grid to form a thin film, which was subsequently counterstained with a 2% phosphotungstic acid solution for 30 seconds. The prepared thin films were allowed to air-dry for 30 minutes at ambient temperature before being examined using TEM (7800HT, Hitachi, Japan).

For the evaluation of storage stability, samples were preserved in PBS or PBS containing 10% FBS at either 4 °C or 37 °C. Particle size measurements were conducted at specific time intervals (1, 3, 5, and 7 days). The in vitro drug release behavior of HA1@diABZI-HMGB1 was assessed using the dialysis bag method. In brief, 1 mL aliquots of HA1@diABZI-HMGB1 were placed into dialysis bags (molecular weight cut-off = 3500 Da, dimensions: 25 mm × 5 m; Spectrum Medical Industries Inc., USA). The sealed bags were then immersed in various fresh dissolution media (PBS, pH 7.4; and citrate buffer, pH 5.5; 50 mL each) maintained at 37 °C and stirred at 100 rpm. At predetermined time points (0.5, 1, 3, 6, 12, 24, 36, 48, 60, 72, 84, 96, 108, and 120 hours), 1 mL samples were withdrawn and immediately replaced with an equivalent volume of preheated dissolution media. The quantities of released diABZI were determined using the previously described HPLC method, and in vitro cumulative drug release profiles were plotted. The cumulative release ratio of diABZI was calculated using the following equation:$\text{Cumulative}\text{ release (\%)}\text{ }\text{ = (}{\text{ }\text{V}}_{S}\sum_{1}^{n-1} \text{C}_{\text{ i }}\text{+}{\text{ }\text{V}}_{\text{0}}\text{C}_{\text{ n }})/m\times100\text{\%}$, where V_s_ is the sampling volume (1 mL); V_0_ is the total volume of the release medium (50 mL); C_i_ is the diABZI concentration in the release medium at each previous sampling point; C_n_ is the diABZI concentration in the release medium at the n-th time point; and m represents the total amount of diABZI initially loaded in HA1@diABZI-HMGB1 added to each dialysis bag. The cumulative release profiles of diABZI were plotted as a function of time.

**Antitumor Immunity Effect *In Vivo***

In order to examine the infiltration of various immune cells within the tumor microenvironment, mice were euthanized three days following the conclusion of the treatment regimen. Tumor tissues were excised and promptly rinsed in cold RPMI 1640 medium supplemented with antibiotics and 1–2% FBS to eliminate residual surface blood. The tissues were subsequently minced into small fragments, approximately 1-2 mm³ in size, and transferred into 50 mL centrifuge tubes. Enzymatic digestion was conducted using a freshly prepared digestion cocktail comprising 1 mg/mL Collagenase Type 4 (Worthington Biochemical), 0.5 mg/mL Collagenase Type 1 (Worthington Biochemical), and 1 mg/mL DNase I (Roche) dissolved in RPMI 1640. The mixture was incubated at 37°C with continuous agitation at 150 rpm for 40 minutes. To facilitate efficient tissue dissociation, the suspension was mechanically disrupted by gentle pipetting (20 repetitions) every 15 minutes using a 1 mL pipette tip with the tip removed. The digestion process was halted by the addition of an equal volume of cold RPMI 1640 supplemented with FBS. The resultant cell suspension was filtered through a 70 μm cell strainer to remove debris, centrifuged at 500 g for 10 minutes, and subsequently resuspended in PBS containing 1% BSA for further analysis. Cells were collected and subjected to washing with FACS buffer. Cell viability was evaluated through staining with Ghost Dye Violet 540 (Tonbo Biosciences) for 20 minutes at room temperature under dark conditions, followed by blocking with anti-CD16/32 for 20 minutes at 4 °C. For surface staining, cells were incubated with fluorochrome-conjugated surface antibodies at 4 °C for 30 minutes in the dark. The antibodies utilized included: BV786 anti-CD45 (Invitrogen, 417-0451-80), PE anti-CD11b (Invitrogen, 12-0112-81), APC anti-F4/80 (Elabscience, E-AB-F0995E), eFluor 450 anti-CD86 (Invitrogen, 48-0862-82), FITC anti-CD11c (BioLegend, 117305), BV711 anti-Ly-6C (BioLegend, 128037), PerCP-eFluor 710 anti-CD3 (Invitrogen, 46-0032-82), BV421 anti-CD4 (Invitrogen, 404-0042-80), and PE/Cyanine7 anti-CD8a (BioLegend, 100722). For panels necessitating intracellular detection, cells were fixed and permeabilized using the Foxp3/Transcription Factor Staining Buffer Set (Thermo Fisher Scientific, 00-5523) in accordance with the manufacturer's instructions. Subsequently, cells were incubated with Alexa Fluor 647 anti-FOXP3 (BioLegend, 320014) and BV650 anti-CD206 (BioLegend, 141723) in permeabilization buffer for 60 minutes at room temperature.

To analyze spleen immune cells, the spleen was gently ground with PBS on ice, and the suspension was filtered through a 70-micron filter to isolate single cells. After removing red blood cells, cell viability was assessed using Ghost Dye Violet 540 for 20 minutes at room temperature in the dark. Cells were then blocked with anti-CD16/32 for 20 minutes at 4°C and incubated with fluorochrome-conjugated surface antibodies for 30 minutes at 4°C in the dark. The following antibodies were utilized in this study: BV786 anti-CD45 (Invitrogen, 417-0451-80), PE anti-CD11b (Invitrogen, 12-0112-81), APC anti-F4/80 (Elabscience, E-AB-F0995E), eFluor 450 anti-CD86 (Invitrogen, 48-0862-82), FITC anti-CD11c (BioLegend, 117305), BV711 anti-Ly-6C (BioLegend, 128037), PerCP-eFluor 710 anti-CD3 (Invitrogen, 46-0032-82), PE/Cyanine7 anti-CD8a (BioLegend, 100722), APC anti-CD62L (BioLegend, 104412), and BV421 anti-CD44 (BioLegend, 103039). For panels necessitating intracellular detection, cells were fixed and permeabilized using the Foxp3/Transcription Factor Staining Buffer Set (Thermo Fisher Scientific, 00-5523) in accordance with the manufacturer’s instructions. Subsequently, the cells were incubated with BV650 anti-CD206 (BioLegend, 141723) in permeabilization buffer for 60 minutes at room temperature.

Following three washes with FACS buffer, the stained cells were assessed using a URIT BF-730 flow cytometer (Guilin Urit Medical Electronic Co., Ltd, China) and subsequently analyzed with FlowJo software. The gating strategies are presented in supplementary Figures 2 and 3. Specifically, M1-like macrophages were defined as CD45^+^CD11b^+^F4/80^+^CD86^+^CD206^-^, M2-like macrophages as CD45^+^CD11b^+^F4/80^+^CD86^-^CD206^+^, dendritic cells as CD45^+^CD11b^+^CD11c^+^, mature dendritic cells as CD45^+^CD11b^+^CD11c^+^CD86^+^, CD8+T cells as CD45^+^CD3^+^CD8^+^, regulatory T cells as CD45^+^CD3^+^CD4^+^Foxp3^+^, MDSC-like myeloid cells as CD45^+^CD11b^+^Ly-6C^+^, and splenic effector memory CD8+T cells as CD45^+^CD3^+^CD8^+^CD44^hi^CD62L^low^.

***In Vivo* Imaging**

To evaluate the targeting capability of the aptamer HA1, Cy5-labeled HA1 and a ssDNA library (5 nmol/100 μL) were administered intravenously to mEC25 tumor-bearing mice. One hour post-injection, the distribution of HA1 was visualized using a live imaging system (X5, Tanon). Subsequently, major organs, including the heart, liver, spleen, lungs, kidneys, and tumor, were excised and further examined using the same imaging system. PBS was used as the control group. To evaluate the in vivo biodistribution of HA1@diABZI-HMGB1, DiR-labeled EYLNs-diABZI, DiR-labeled HA1@diABZI, DiR-labeled EYLNs-diABZI-HMGB1, and DiR-labeled HA1@diABZI-HMGB1 were prepared and administered intravenously to mEC25 tumor-bearing mice. Live imaging was conducted at predetermined intervals (1, 3, 6, 9, 12, and 24 hours). Following the final imaging session, the mice were euthanized, and tumors along with major organs were collected to visualize the distribution patterns across different treatment groups. Fluorescence signals were quantified by region-of-interest (ROI) analysis using the imaging analysis software. Before imaging, tumor-bearing mice were depilated to reduce hair-associated autofluorescence and light scattering. For in vivo imaging, ROIs were drawn over the tumor regions, and background ROIs of the same size were placed in adjacent non-tumor regions of the same mouse. Background-corrected fluorescence intensity was calculated by subtracting the background ROI signal from the tumor ROI signal. For ex vivo imaging, ROIs were drawn to cover the entire excised tumor or organ, and background correction was performed using ROIs placed in non-fluorescent regions of the same image. All images within the same experiment were acquired under identical imaging parameters. The number of mice analyzed at each time point was 3 per group.

**Detection of peripheral blood circulation behavior**

Mice bearing mEC25 tumors were administered various treatments, including DiR-labeled EYLNs-diABZI, DiR-labeled HA1@diABZI, DiR-labeled EYLNs-diABZI-HMGB1, and DiR-labeled HA1@diABZI-HMGB1. Peripheral blood samples were collected at 1, 3, 12, and 24 hours post-injection and subsequently centrifuged to remove erythrocytes. The resulting blood samples were then transferred to 96-well plates for the assessment of fluorescence intensity via in vivo imaging.

**H&E, Ki67 and TUNEL assays**

Hematoxylin and eosin (H&E) staining was employed to differentiate between normal and pathological tissues. Specifically, 5 μm tissue sections were procured from tumor samples. These sections underwent deparaffinization, rehydration, and rinsing as previously outlined in the literature. Subsequently, the sections were stained with H&E, dehydrated through a graded alcohol series, cleared in xylene, and mounted with a neutral resin. Ki-67, a marker indicative of cell proliferation and cell cycle phase distribution, was utilized for immunostaining. The tissue sections were incubated with an anti-Ki67 antibody, followed by incubation with a goat anti-rabbit peroxidase-conjugated secondary antibody. Staining was completed using 3,3-diaminobenzidine substrate and hematoxylin. TUNEL staining was performed using a TUNEL staining kit to label DNA strand breaks. The sections were incubated with proteinase K for 15 minutes at 37 °C, followed by incubation with the TUNEL reaction solution. Apoptotic cells were visualized via green fluorescence, and cell nuclei were counterstained with DAPI. Histological images were captured using an upright fluorescence microscope (Leica, USA).

**Hemolysis test**

Peripheral blood was collected from C57BL/6 mice and subjected to centrifugation at 1500 g for 10 minutes at 4 °C to isolate red blood cells (RBCs). The RBCs were subsequently resuspended in PBS and combined with varying concentrations of HA1@diABZI-HMGB1. Following a 4-hour incubation period at 37 °C, the supernatant was analyzed for OD450nm to assess the hemolysis rate. In this assay, 1% Triton-X 100 and PBS were utilized as positive and negative controls, respectively.


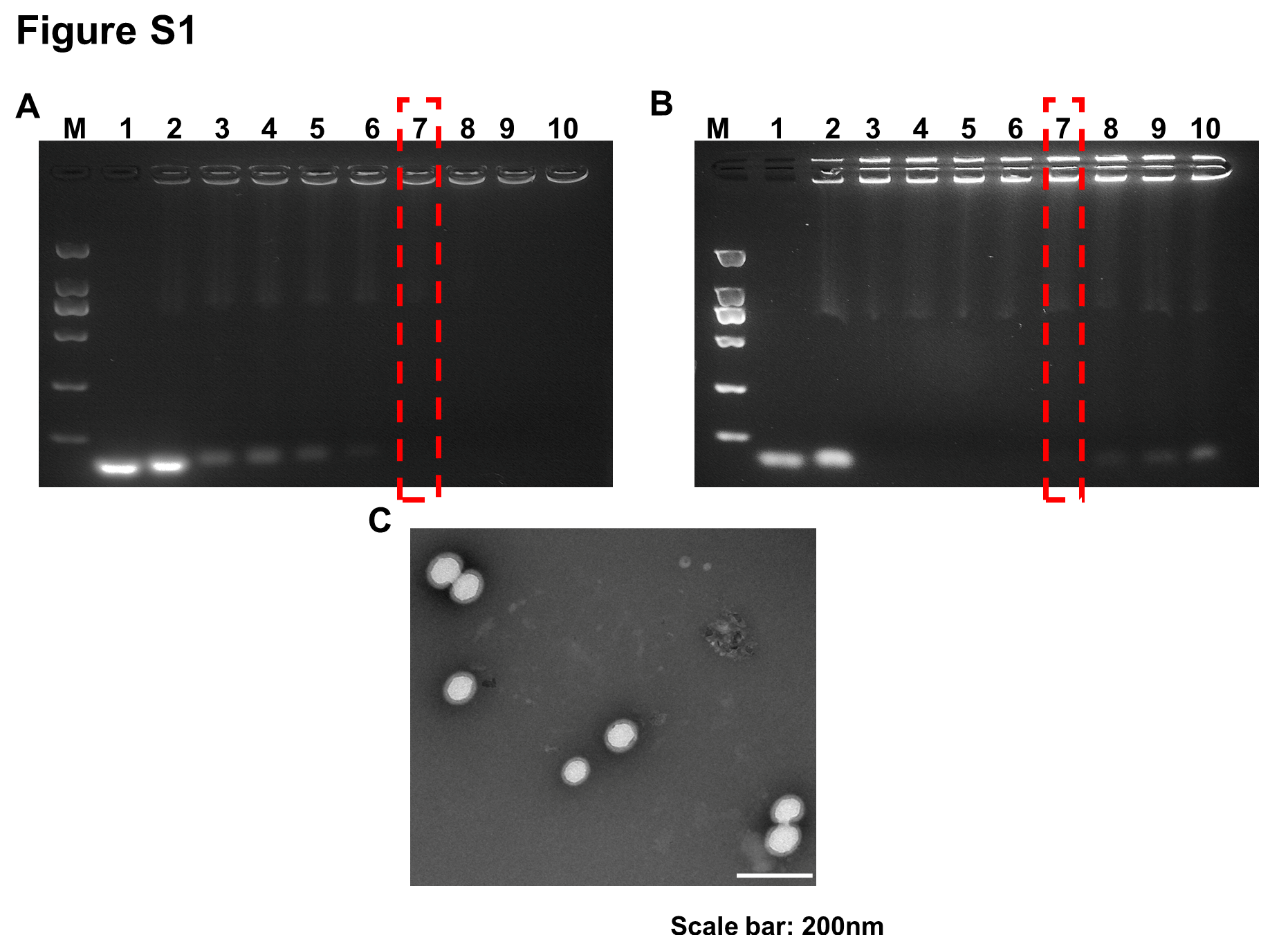


**Figure S1. Optimization of the formulation of HA1-modified EYLNs and transmission electron microscopy characterization of HA1@diABZI-HMGB1.**

**(A)**. 3% agarose gel electrophoresis used to optimize the amount of polyethyleneimine (PEI) in EYLNs. The ratio was fixed at 3 mg EYLNs: 22.15μgdiABZI: 3 nmol HA1, while different amounts of PEI were added . Lane M, DNA marker; lane 1, free HA1; lane 2, free HA1 + EYLNs-diABZI; lanes 3–10, 8.25, 16.5, 27.5, 41.25, 82.5, 165, 247.5, and 412.5 μg PEI, respectively. **(B)**. 2% agarose gel electrophoresis used to optimize the amount of HA1 conjugated to EYLNs. The ratio was fixed at 3 mg EYLNs: 22.15μg diABZI: 82.5 μg PEI, while different amounts of HA1 were added. Lane M, DNA marker; lane 1, free HA1; lane 2, free HA1 + EYLNs-diABZI; lanes 3–10, 0.5, 1.5, 2.0, 2.5, 3.0, 3.5, 4.0, and 4.5 nmol HA1, respectively. **(C).**Wider-field TEM images of HA1@diABZI-HMGB1. Scale bar: 200nm.


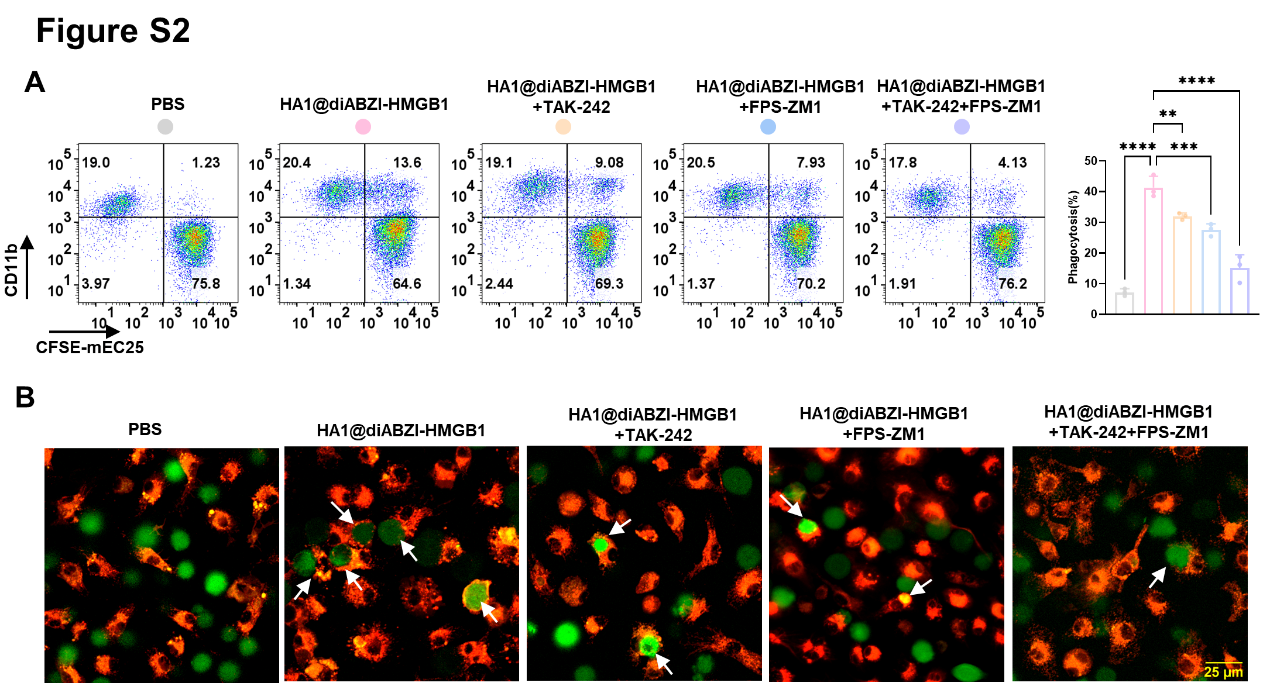


**Figure S2. TLR4 and/or RAGE blockade attenuates HA1@diABZI-HMGB1-associated macrophage phagocytosis.**

**(A).** Representative flow cytometry plots illustrate the phagocytosis of mEC25 tumor cells by bone marrow-derived macrophages (BMDMs) following various treatments. The mEC25 cells were labeled with CFSE, while the macrophages were stained with CD11b. Data are shown as the mean ± SD $n=3$. Statistical significance was calculated through one-way ANOVA using the Tukey’s post-test. **p < 0.01, ***p < 0.001, and ****p < 0.0001. **(B).** Confocal laser scanning microscopy images depict mEC25 tumor cells engulfed by BMDMs after different treatments, with mEC25 cells labeled in green using CFSE and BMDMs labeled in red using PKH26. Scale bar: 25 μm.


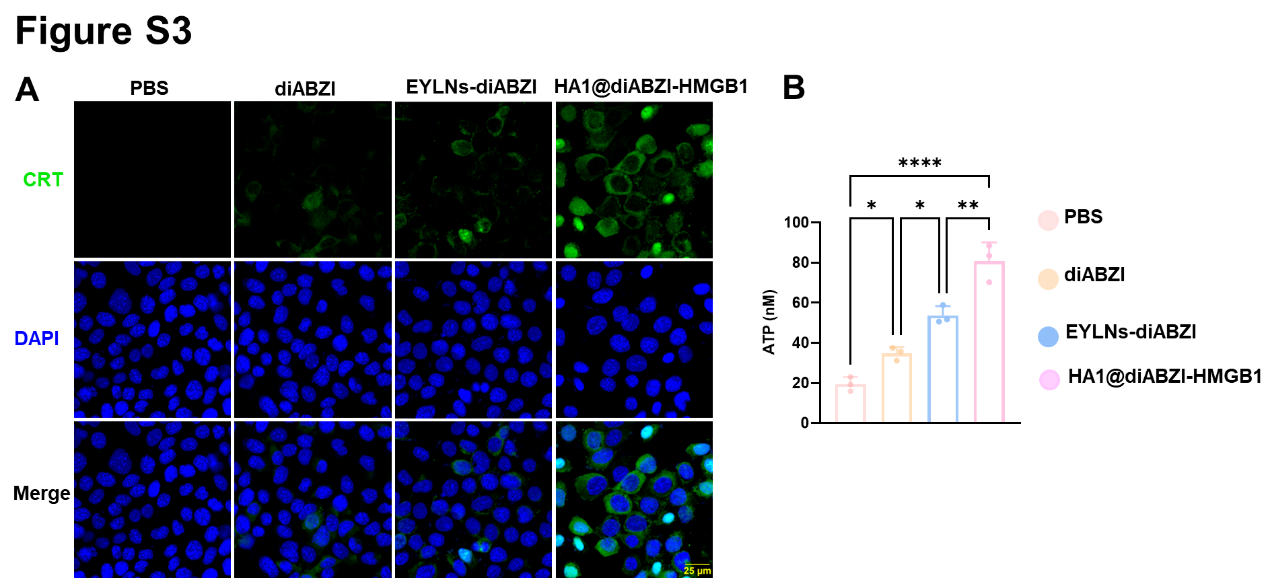


**Figure S3. Detection of classical immunogenic cell death (ICD) markers of tumor cells in each treatment group.**

**(A).** Confocal laser scanning microscopy images of CRT expression in mEC25 tumor cells. Scale bar:25μm; **(B)** Detection of extracellular ATP secretion. Data are shown as mean ± SD (n = 3). Statistical significance was calculated through one-way ANOVA using the Tukey’s post-test. *p< 0.05, **p < 0.01, and ****p < 0.0001.


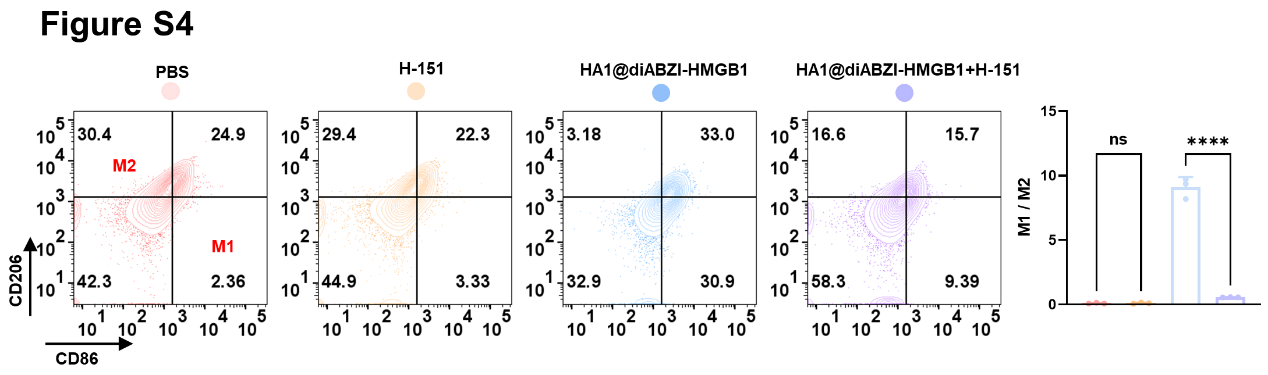


**Figure S4. STING inhibition attenuates HA1@diABZI-HMGB1-induced macrophage repolarization.**

Representative flow cytometry plots showing macrophage phenotypes under the indicated treatments. M2-like BMDMs were pretreated with the STING inhibitor H-151 before stimulation with HA1@diABZI-HMGB1. The proportions of CD11b+F4/80+CD86+CD206- M1-like macrophages and CD11b+F4/80+CD86-CD206+ M2-like macrophages, or the corresponding M1/M2 index, are shown. Data are shown as mean ± SD (n = 3). Statistical significance was calculated through one-way ANOVA using the Tukey’s post-test. ****p < 0.0001.


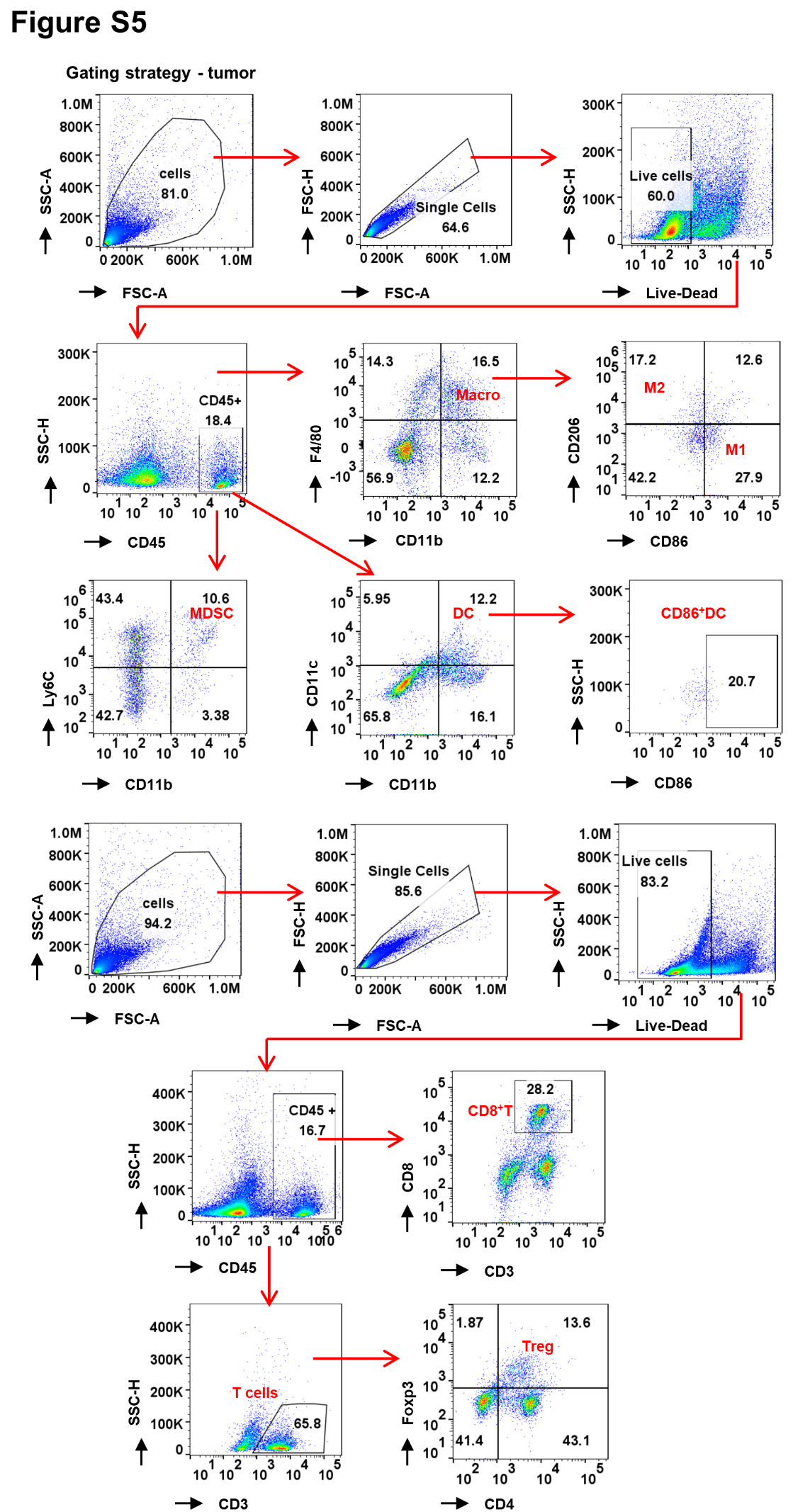


**Figure S5. The gating strategies for examining the infiltration of various immune cells within the tumor microenvironment.**


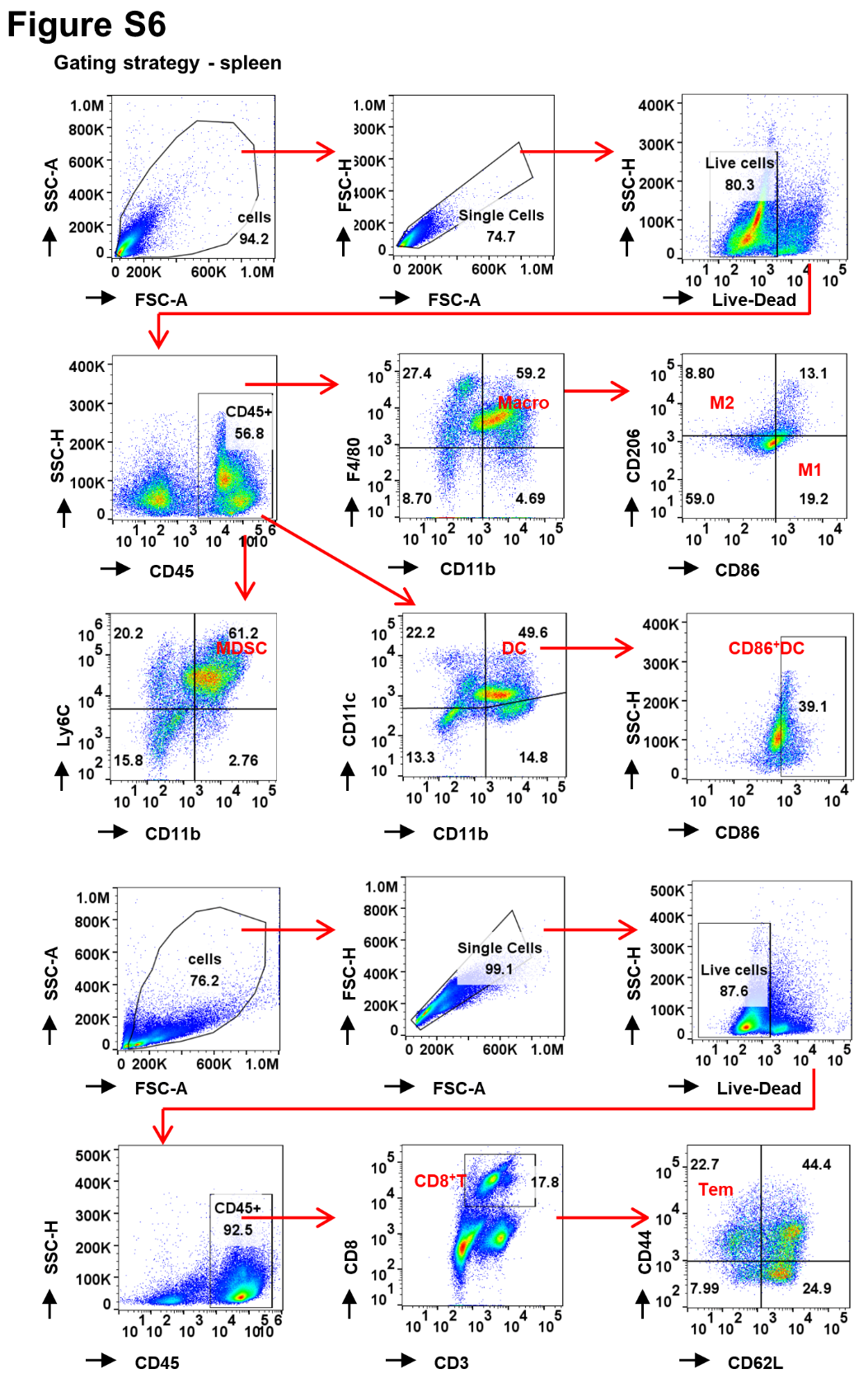


**Figure S6. The gating strategies** **employed for gating strategies in the analysis of splenic immune cells.**
